# A cerebellar granule cell–climbing fiber computation to learn to track long time intervals

**DOI:** 10.1016/j.neuron.2024.05.019

**Published:** 2024-06-12

**Authors:** Martha G. Garcia-Garcia, Akash Kapoor, Oluwatobi Akinwale, Lina Takemaru, Tony Hyun Kim, Casey Paton, Ashok Litwin-Kumar, Mark J. Schnitzer, Liqun Luo, Mark J. Wagner

**Affiliations:** 1 National Institute of Neurological Disorders & Stroke, National Institutes of Health, Bethesda, MD 20894, USA.; 2 Department of Biology and Howard Hughes Medical Institute, Stanford University, Stanford, CA 94305, USA.; 3 Department of Applied Physics, Stanford University, Stanford, CA 94305, USA.; 4 Zuckerman Mind Brain Behavior Institute, Department of Neuroscience, Columbia University, NY 10027, USA.; 5 These authors contributed equally to this work.; 6 Lead Contact

## Abstract

In classical cerebellar learning, Purkinje cells (PkCs) associate climbing fiber (CF) error signals with predictive granule cells (GrCs) active just prior (~150ms). Cerebellum also contributes to behaviors characterized by longer timescales. To investigate how GrC-CF-PkC circuits might learn seconds-long predictions, we imaged simultaneous GrC-CF activity over days of forelimb operant conditioning for delayed water reward. As mice learned reward timing, numerous GrCs developed anticipatory activity ramping at different rates until reward delivery, followed by widespread time-locked CF spiking. Relearning longer delays further lengthened GrC activations. We computed CF-dependent GrC→PkC plasticity rules, demonstrating that reward-evoked CF spikes sufficed to grade many GrC synapses by anticipatory timing. We predicted and confirmed that PkCs could thereby continuously ramp across seconds-long intervals from movement to reward. Learning thus leads to new GrC temporal bases linking predictors to remote CF reward signals—a strategy well-suited to learn to track long intervals common in cognitive domains.

## eTOC

Via the first simultaneous recordings of cerebellar granule cell and climbing fiber activity, Garcia-Garcia et al. demonstrate a new type of cerebellar computation suited to learning to track long time intervals via sustained neural activity ramping. This strategy could enable cerebellar contributions to longer-timescale cognitive processes requiring temporal learning.

## Introduction

**Figure 1 |:**
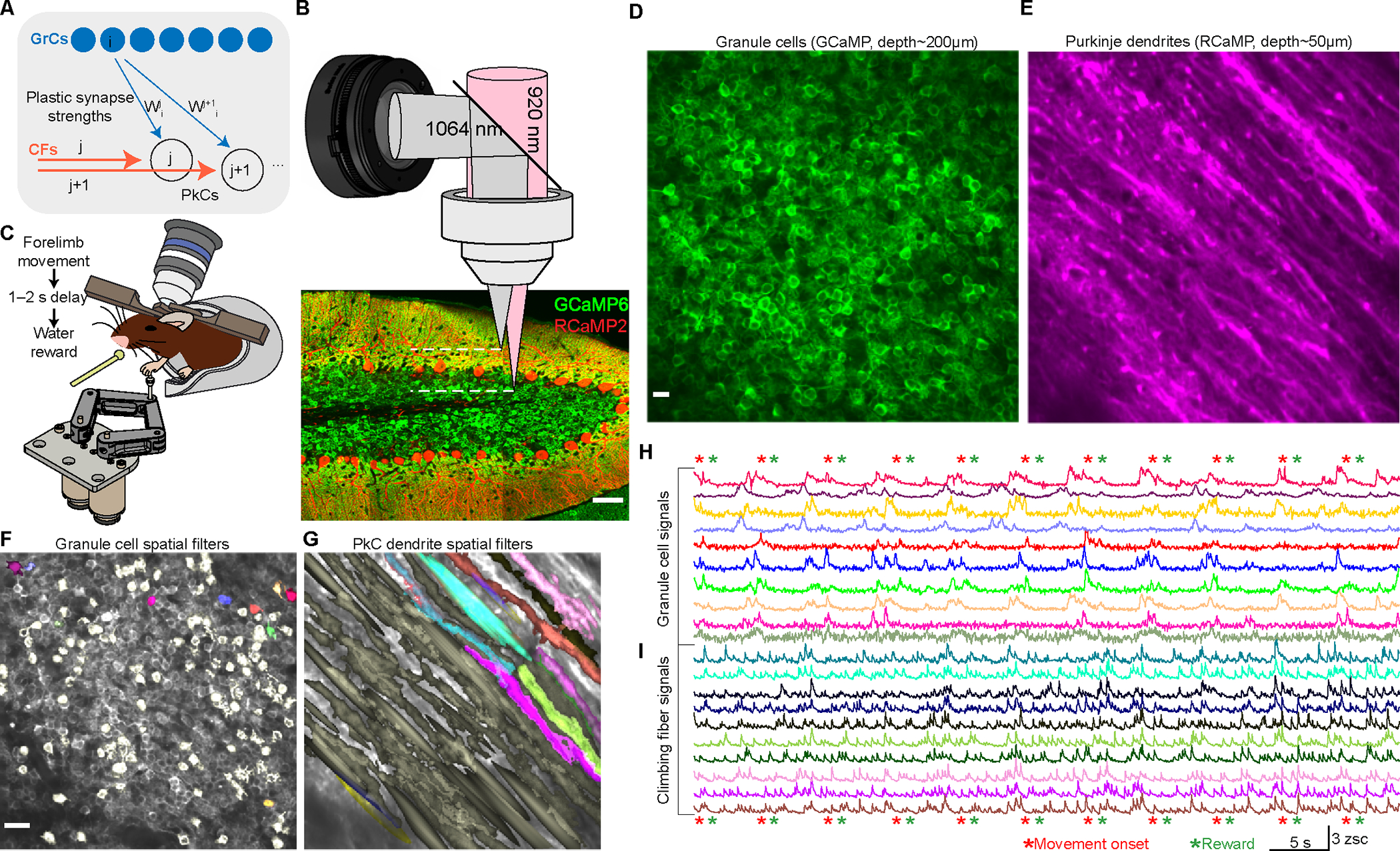
Two-color, two-depth, two-photon Ca^2+^ imaging of cerebellar GrCs and CFs. **(A)** Schematic of cerebellar microcircuit: ~100,000 GrCs and one CF innervate each PkC, with GrC→PkC synapses adjusted by CF-dependent plasticity. GrC “i” synapses on PkCs “j” and “j+1” with weights W_i_^j^ and W_i_^j+1^. **(B)** Imaging schematic and histology. GrCs transgenically expressed GCaMP6f while PkCs virally expressed R-CaMP2. PkC dendritic Ca^2+^ reports complex spikes^44^ and thus CF activity. Through one objective, a 920 nm laser excited GCaMP in GrC somas while a remotely-focused 1064 nm laser excited PkC dendrites. **(C)** Mice grasped a robotic manipulandum and self-initiated 8-mm-maximum forward pushes of at least 6–7 mm for water reward following a delay (main GrC-CF data: 1.1 s; all other studies: 1-s or 2-s). 2-s after reward time, the handle automatically returned over the following 2-s. **(D,E)** Example *in vivo* mean two-photon simultaneous images of GrCs (**D**) and PkC dendrites (**E**). **(F,G)** Example extracted GrCs (**F**) and PkC dendrites (**G**). Detected spatial filters of active cells are superposed in pale yellow, or for 10 GrCs and PkC dendrites in colors corresponding to the traces in **H,I**. **(H,I)** For 10 GrCs (**H**) and PkC dendrites (**I**), color-matched to cells in (**F**) and (**G**), traces show time-varying fluorescence of each neuron. Stars show forelimb movements and rewards. PkC dendritic spiking hereafter referred to as CFs.

The cerebellum is widely viewed as a structure for learning predictions^1–5^ from inputs ranging from the body to neocortical cognition centers^6–9^. These diverse inputs propagate through a uniform circuit (Figure 1A): PkCs receive input from ~100,000 GrCs^10^ and just one CF. PkC computation depends on integrating GrC inputs^11,12^ using synaptic strengths that can be modified by CF instructive signals^13^. Specifically, when a PkC receives a CF spike, any GrC inputs active in the preceding ~150 ms are weakened via long-term depression (LTD)^14–19^. Similarly, reduced CF activity triggers potentiation (LTP) on coincidently active GrC inputs^20–22^. Given the brief plasticity window to “sense” active GrCs, a core cerebellar function is learning short-latency associations between predictive events and CF errors^23–27^. Thus, decades of theory have posited GrC representations of time, or “basis sets,” as key to cerebellar learning^28–36^. Yet limited available data suggests varied types of GrC representations^37–41^. Instead of a single uniform GrC basis, an intriguing alternative is that GrCs assume differing profiles to suit specific computational needs. In this scenario, understanding cerebellar computations for different learned behaviors would require characterizing the associated GrC bases and their relationship to CF teaching signals.

Cognitive behaviors including those with cerebellar contributions^4,42,43^ often require linking events separated by seconds or more. Cerebellar computations in such contexts remain obscure, and the associated GrC bases and their interplay with CF teaching signals are uncharacterized. Due to technical difficulty, GrC and CF inputs onto PkCs have yet to be simultaneously observed and related to learning.

Here we recorded simultaneous GrC and CF activity using dual-color two-depth two-photon Ca^2+^ imaging over days of operant conditioning for delayed reward. Mice learned to lick in anticipation of expected reward—a behavior with cerebellar contributions. Meanwhile, GrCs and CFs developed two key features. First, after learning, numerous GrCs ramped activity up and down at differing rates from forelimb movement until reward 1 s later; subsequent relearning of a 2-s delay further lengthened GrC profiles commensurately over days. Second, reward itself triggered widespread time-locked CF spiking that persisted throughout learning. To understand the computations these GrC-CF patterns subserve, we predicted GrC→PkC synaptic changes based on canonical plasticity rules. Because many GrCs developed activity that spanned the delay, CF-driven plasticity near reward sufficed to grade numerous GrC→PkC weights by GrC activity timing throughout the delay. We therefore predicted that PkC spiking could adapt to track the interval from forelimb movement until reward up to 2-s later. PkC recordings confirmed such delay-spanning activity ramps. Thus, the emergence during learning of new GrC bases, which link predictive events to reward-evoked CF teaching signals seconds later, permits long-timescale associations. This outlines a cerebellar computation suited to learning neural ramps that track long intervals common in cognitive domains.

## RESULTS

### Simultaneous GrC-CF recordings during reward-driven learning

We sought to simultaneously record GrC and CF activity over learning, which has previously been impossible. Due to tight packing and small cell size, two-photon imaging is necessary to track mammalian GrC populations^9,40,41^. Two-photon imaging can also record CF activity via PkC dendritic Ca^2+^, which reports complex spikes^44^. Thus, in principle, GrCs and CFs could simultaneously be imaged in a single-color. However, because ~100,000 GrC axons physically intersect each PkC dendrite, it would be impossible to spatially disambiguate their fluorescence *in vivo*. Instead, we leveraged spectral separation, via dual-color imaging.

Cerebellar areas related to cognition include Lobules ~VI-VIII^4^. Lobule VI is linked to multiple cognitive circuits^45–49^, exhibits nonmotor signaling^9,41^, and receives most inputs to its GrCs from pons^50^, which relays neocortical information. While these features are common in cerebellum, to deeply characterize GrC-CF computation in one region, we centered our imaging on LVI right vermis (ipsilateral to the reaching forelimb). We transgenically expressed GCaMP6f in GrCs (Math1-Cre/Ai93/ztTA^41^), and virally expressed R-CaMP2^51^ in PkCs (AAV–L7–6^52^–R-CaMP2) (Figure 1B, S1A histology), whose dendritic Ca^2+^ reliably reports complex spikes and thus CF input^44,53^. Next, to simultaneously visualize both, we imaged at two depths: a 920 nm laser illuminated GrC somas ~150–300 μm below the brain surface, while a remotely-focused 1064 nm laser illuminated PkC dendrites at a depth of ~50 μm (Figure 1B).

While animals learned an operant behavior (Figure 1C; details below), we simultaneously monitored GrCs (143±5 per session, 117 sessions in 20 mice) and PkC dendrites (hereafter referred to as CFs; 55±2 PkC dendrites per session), either at 30 Hz or 22.5 Hz (example: Figure 1D,E; Video S1), over 7.5±0.4 days (Figure S1B–G). We extracted spatial locations (Figure 1F,G) and time-varying fluorescence (Figure 1H,I) of each neuron, and further extracted spike times for CFs^44^ (**Methods**). Importantly, because we imaged GrCs directly below the imaged PkC dendrites (field of view: ~235×235 μm^2^), axons of these GrCs likely physically intersected most PkC dendrites, and GrCs synapse onto ~50% of PkC dendrites they intersect^54^ (Figure S1H,I). Thus, we visualized both input streams—CF and some of the GrCs—to the same dozens of PkCs over learning.

**Figure 2 |:**
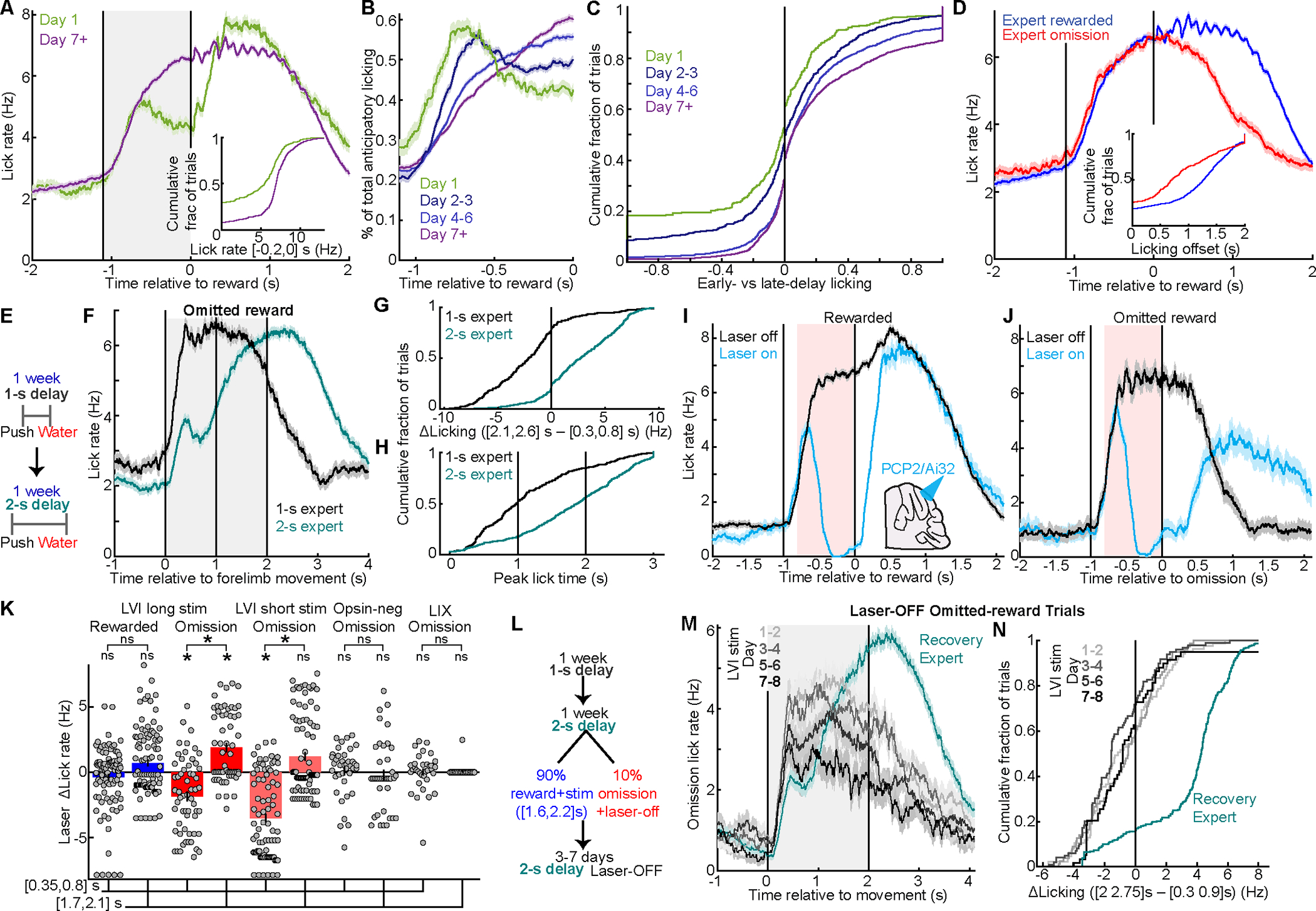
Mice learn to elevate licking near the time of expected reward, with cerebellar contributions **A,** Lick rate before reward was higher on Day 7+ than Day 1 (334 Day 1 trials from 10 mice and 1,570 Day 7+ trials from 20 sessions in 12 mice; this and subsequent grey regions denote delay period and vertical lines denote movement and reward). Inset, p<10^−6^. **B,** Distribution of licks across delay (400/9, 919/16, 1,820/21, and 2,065/22 trials/sessions). **C,** Novice mice licked more early than late in the delay, whereas expert mice inverted this pattern ((late − early)/(late + early). Early: [−0.8, −0.6] s, late: [−0.2, 0] s. p<10^−6^). These and all subsequent centers denote means and shaded regions and error bars denote SEM across observations (see also Table S1). **(D)** Expert lick rate during rewarded and omitted reward trials (1,570 and 495 trials from 20 sessions in 12 mice). Inset, last time (max 2 s) at which lick rate exceeded 70% of the prior peak ([−0.25,0.5] s; p<10^−6^; 0 if licking never fell below 50% of peak). **(E-H)** Some mice trained with a 1-s delay followed by a 2-s delay (E). F, Licking on omitted reward trials for 1-s vs 2-s experts (283 1-s and 519 2-s trials from 14 mice, 16 sessions each). (G) 2-s expert omission licking was higher after 2-s than early in the delay, while 1-s experts showed the opposite pattern (p<10^−6^). (H) Omission licking peaked near respective expected reward times (p<10^−6^, peak over [0,3] s). **(I-N)** PCP2Cre/Ai32 PkC stimulation studies. **(I,J)** PCP2Cre/Ai32 mice with windows over right cerebellum LVI trained on the 1-s-delay task. Starting 0.2 s after mice pushed >7-mm, we activated ChR2 for 0.8 s on 20–40% of trials (I, Rewarded: 478 laser-off, 99 laser-on; J, omitted reward: 68 laser-off, 59 laser-on; 7 mice). Stimulation abolished anticipatory licking. Reward triggered recovery of normal licking, but on omission trials licking remained weaker and less well-timed (controls, Figure S2J–L). Pink: mean laser period. **(K)** Quantification of Figure 2I,J, S2J,K,L. “ΔLick rate”: each laser-on trial’s licking minus the mean laser-off licking, averaged from [0.35,0.8] s or [1.7,2.1] s from reward. Rewarded licking (Figure 2I) changed little in either window (p=0.8 and 0.06; difference p=0.1). Omission licking was reduced just after reward time (p=3×10^−5^ and <10^−6^ for long/Figure 2J and short/Figure S2J stim paradigms), but aberrantly elevated later (long stim: p=3×10^−5^; brief-stim, p=0.06; both paradigms late vs early p<10^−6^). Opsin-negative and LIX controls were not significant (bars p=0.2, 0.4, 0.3, 1; differences p=0.1, 0.7; laser-on omitted-reward trial counts: 99, 59, 85, 39, 34; mice/session counts: 7/9, 7/7, 3/3, 3/3). **(L-N),** PCP2-Ai32 mice that were experts on the 1-s delay retrained with 2-s delay, but with PkC stimulation on every rewarded trial from [1.6, 2.2] s from movement (90% of all trials, L). 10% of trials were laser-off reward-omission “probe” trials. Over 1 week of perturbed training—but evaluated on probe laser-OFF trials—mice never learned to lick near the 2-s reward delivery time (M, grayscale curves). During subsequent laser-OFF training, mice learned to lick near 2-s (M, green; 85, 79, 99, 59, and 261 reward omission trials per condition from 7 mice). N, Δlick rate during expected reward time minus early delay (recovery>laser-on p<10^−6^; laser-on across learning p=0.07).

During imaging, water-restricted mice grasped a robotic arm to self-initiate forward pushes (Figure 1C). Following successful movement and a delay (main GrC-CF imaging dataset: 1.1-s, all other studies: 1 or 2 s), animals received water reward. Delays were thus far longer than classical cerebellar association timescales^55^. Mice acclimated to the task for ~1–3 sessions before we started imaging (**Methods**). Thus, “Day 1” hereafter refers to the first imaging session. After ~1–2 imaging sessions, we also withheld water on a random 20% of trials (“omitted reward”). Mice learned the task over ~1 week: (1) movement duration decreased (Figure S2A); (2) execution rate increased (Figure S2B–D); and (3) anticipatory licking increased (Figure 2A)^41,56^.

### Mice learn the timing of delayed reward during operant conditioning

We first tested whether mice learned reward timing. To distinguish the amount of anticipatory licking from its timing, we examined the temporal distribution of licks prior to reward in mice trained on a 1.1-s delay (Figure 2B). Novice mice licked more just after forelimb movement than just before reward, while expert mice partly inverted this pattern (Figure 2C, S2E; late v. early preference: −0.13±0.02 novice vs +0.2±0.01 expert; these and all subsequent quantifications mean±SEM across observations, see also Table S1). On omitted reward trials, experts reduced licking after the expected reward time (Figure 2D, S2F; licking off-time, last time lick rate exceeded 70% of peak). Thus, mice learned to concentrate licking closer to expected reward.

To assay reward timing learning directly, we tested whether behavior adapted to delay duration. Mice trained on a 1-s delay, and then retrained on a 2-s delay for another week (Figure 2E). We examined licking on omitted reward trials aligned to forelimb movement, such that differences after t=0s could not be attributed to exogenous stimuli (Figure 2F, S2G, Video S2). While 1-s experts licked more shortly after forelimb movement compared to after 2-s, 2-s-experts exhibited the opposite pattern (Figure 2G). Moreover, when 1-s-experts initially switched to the 2-s paradigm, their licking was diffuse across the delay, consistent with timing uncertainty (Figure S2H,I). Thus, mice adapted their peak licking to the respective reward times (Figure 2H), demonstrating reward timing learning over delays of 1 or 2-s.

### Cerebellum contributes to execution and learning of expectation-driven licking

To test cerebellar contributions to this behavior, we implanted windows over right LVI in PCP2-cre/Ai32 mice, which express ChR2 in all PkCs^57–59^. Activating inhibitory PkCs suppresses the cerebellar nuclei, thus reducing the circuit’s influence on downstream brain regions. Mice trained for a week on the 1-s-delay operant task. In experts, on a random 20–40% of trials, we activated PkCs at 40 Hz for 0.8 s triggered during the delay period (200-ms after a 7-mm-reach-distance threshold; **Methods**). Stimulation largely abolished anticipatory licking (Figure 2I, S2M, Video S3). After laser offset, reward delivery triggered rapid recovery of licking (Figure 2I). However, after laser offset on reward omission trials, licking remained disrupted throughout the trial (Figure 2J). First, mice recovered less licking in the early post-omission period (Figure 2J,K). Second, long after omission of expected reward, mice exhibited aberrant *elevated* licking (Figure 2J,K). Thus, transient cerebellar perturbation imparted long-lasting disturbance to the amount and timing of expectancy-driven licking, but not reward-evoked licking.

We performed several further controls. First, to test whether mice simply needed more recovery time after stimulation, we repeated the experiment using briefer PkC stimulation early in the delay on half of reward omission trials. Nevertheless, recovery of anticipatory licking remained weak and poorly timed (Figure S2J). We next repeated laser illumination in opsin-negative mice (Figure S2K), and finally we repeated PCP2/Ai32 stimulation in a less relevant region (vermis lobule IX, Figure S2L)—both without effect.

These data suggested that cerebellum contributes to expectation-driven licking, leading us next to test its involvement in learning reward-timed licking. We retrained 1-s-expert PCP2/Ai32 mice on a 2-s delay. 90% of trials were rewarded, but also laser-ON ([1.6,2.2] s from movement); 10% of trials were laser-OFF reward-omission probe trials (Figure 2L). Over 1-week of training, licking on probe laser-OFF reward-omission trials never adapted to the 2-s reward time (Figure 2M,N). After 3–7 subsequent days of 100% laser-off training, mice learned to lick correctly. These results suggest that posterior cerebellar cortex contributes to learning and execution of licking driven by expectation of upcoming reward, but do not exclude similar effects for other regions untested in the present study.

### GrCs ramp from movement until reward, which triggers time-locked CF spiking

**Figure 3 |:**
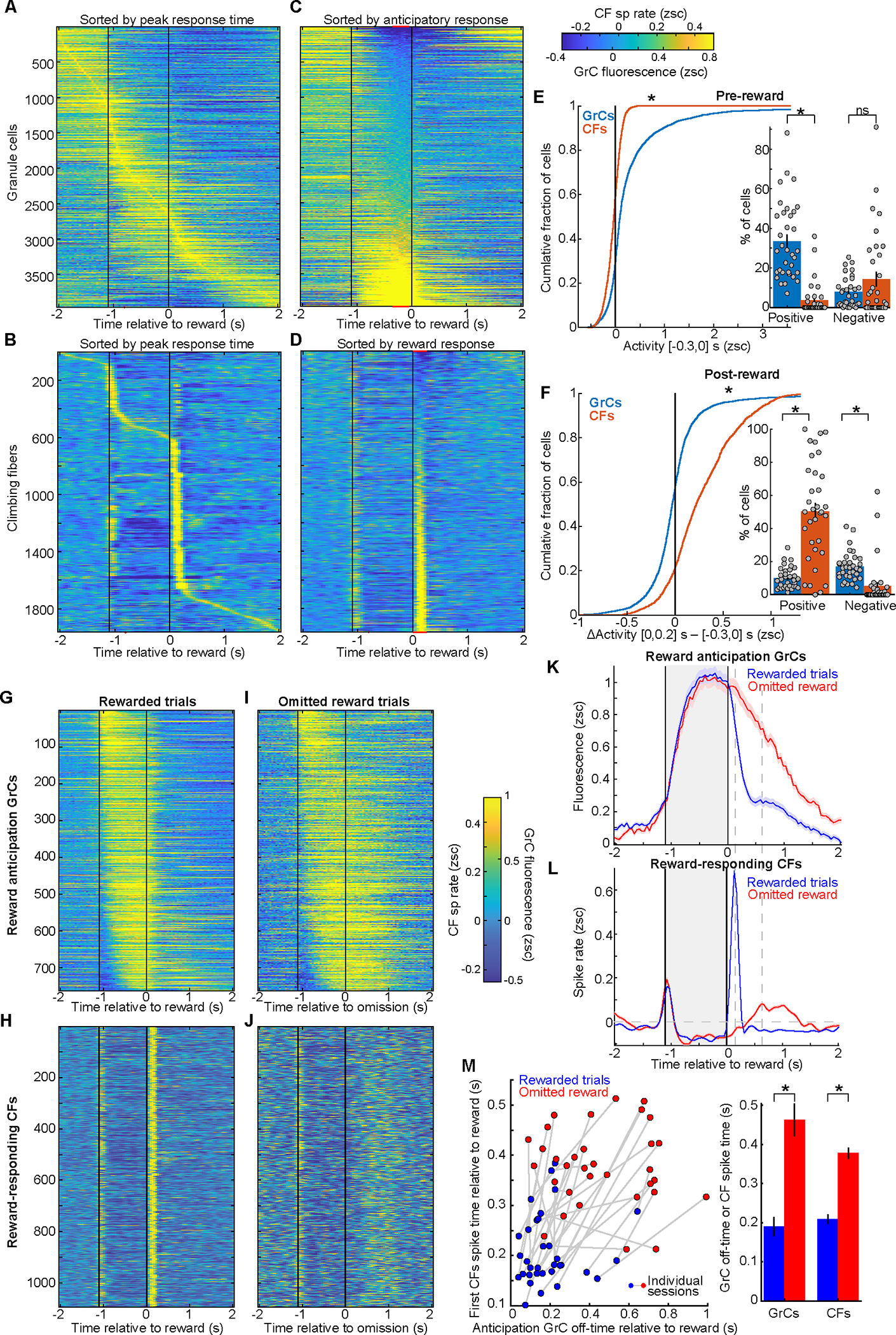
Reward-anticipating GrC activity followed by reward-evoked CF spiking in expert mice **(A-D)** Each row shows the fluorescence (GrCs, A and C) or spike rate (CFs, B and D) of a single neuron, aligned to reward delivery and averaged across expert rewarded trials (3,965 GrCs, 1,964 CFs, 34 sessions/20 mice). Cells sorted by time of peak activity (A,B) or magnitude of anticipatory (C) or reward-evoked activity (D). Red lines denote sorting quantification windows. **(E,F)** Activity quantifications either during delay (E) or post-reward (F), shown as histograms and (insets) binary statistical categories (p<0.05 and magnitude exceeding ±0.2 z-scores (“zsc”)). *p<10^−6^. ns, p=0.54. **(G-J)** Rasters of anticipatory GrCs (G,I) and reward-activated CFs (H,J) on rewarded (G,H) or omitted reward trials (I,J, 20% omissions). 762 GrCs spread across 33/34 expert sessions in 19/20 mice; >0.1 zsc comparing [0.3 –0.03] s to both [−1.3 −1] s and [+0.3 +0.5] s; 1094 CFs spread across all 34 expert sessions/20 mice; spike rate during [0 0.2] s >0.1 zsc, and >0.1 zsc higher than pre-reward [−0.3 −0.03] s). See also Figure S2N,O. **(K,L)** For neurons in (G-J), averages across trials and cells. Grey lines show times of first CF peaks following reward delivery and omission respectively. **(M)** Dots show mean across trials of GrC anticipatory off-time (x-axis, when fluorescence fell to <50% of peak over [-0.5 0] s), versus time of first population CF spiking (y-axis, when average rose above 20^th^ percentile of the reward response [0 0.2] s). From sessions contributing to (G-J), 33/34 sessions, 19/20 mice, on trials with elevated CF reward spiking (117±6 trials per session). Inset, Average across sessions (p=1.6×10^–6^ for both, 33 sessions).

To examine GrC-CF task representations in 1.1-s-delay experts, we computed average activity on rewarded trials (Day 7+) (Figure 3A,B). We noted numerous GrCs with sustained activity during the delay, and many CFs active just after reward (Figure 3C,D). Quantitatively, cells active during the delay composed 34±3% GrCs but only 4±1% of CFs (Figure 3E). Conversely, cells that activated at reward composed 50±5% of CFs but only 10±1% of GrCs (Figure 3F). A smaller 22±5% of CFs activated at movement (Figure S2P). In addition to CF spiking after reward, we also observed modest but variable suppression of CFs prior to reward (14±4% of CFs, Figure 3F). Experts thus featured widespread sustained GrC delay activity and CF reward spiking.

Many GrCs and CFs appeared linked with anticipating and receiving reward. To characterize these features, we identified “reward anticipation” GrCs with elevated delay activity (Figure 3G, S2N,O. 19% of GrCs, from 33/34 sessions in 19/20 mice). Similarly, we identified CFs with reward spiking (Figure 3H, 55% of CFs, from all 34 sessions/20 mice). This revealed that whereas GrC anticipation terminated after reward, it was prolonged after unexpected reward omission (Figure 3I). CF reward spiking was absent on omission trials (Figure 3J). Instead, CF spiking rose later after reward omission, grossly coinciding with the delayed off-time of GrC anticipation (Figure 3K,L). To quantify, for each trial, we computed the times after reward (or omission) when (1) CF spiking rose, and (2) GrC anticipation terminated; both increased significantly on omitted reward trials (Figure 3M). Thus, these GrC-CF phenomena likely relate first to anticipation and then either reward delivery or recognition of its omission.

Instead of signaling reward anticipation, could GrCs simply signal licking? When animals awaited reward, both anticipatory GrC signals and anticipatory licking ramped together. However, GrC activity otherwise diverged from licking: (1) after reward delivery, GrC anticipation *terminated* (Figure S3A, decay time 0.19 s post-reward) – yet licking *further increased* to its highest values (Figure S3A; decay time 1.1 s post-reward); (2) compared to *rewarded* trials, following reward *omission* GrC anticipation was substantially more *prolonged* (Figure S3B, signals extended by 130%) – yet licking was substantially *weaker* and more *brief* (Figure S3B, 37% less licking). Finally, anticipatory GrCs were not modulated by individual lick onsets (Figure S3C,D). We also did not observe selective delay period body, eye, or whisker movements consistent with anticipatory GrC temporal profiles (Figure S3J–Q). Thus, anticipatory GrC signals were most consistent with a temporally building expectation of reward.

Overall, we found that reward anticipation: (1) followed forelimb movement and modest CF spiking; (2) rose with sustained GrC activation; and (3) terminated at reward (or omission) when GrC anticipation decayed and many CFs spiked. This hinted that many expert GrCs spanned the delay from movement until reward-evoked CF spiking. Thus, we next examined whether this was linked to learning reward timing.

### Learning increases GrC reward anticipation, but leaves reward-evoked CF spiking unchanged

**Figure 4 |:**
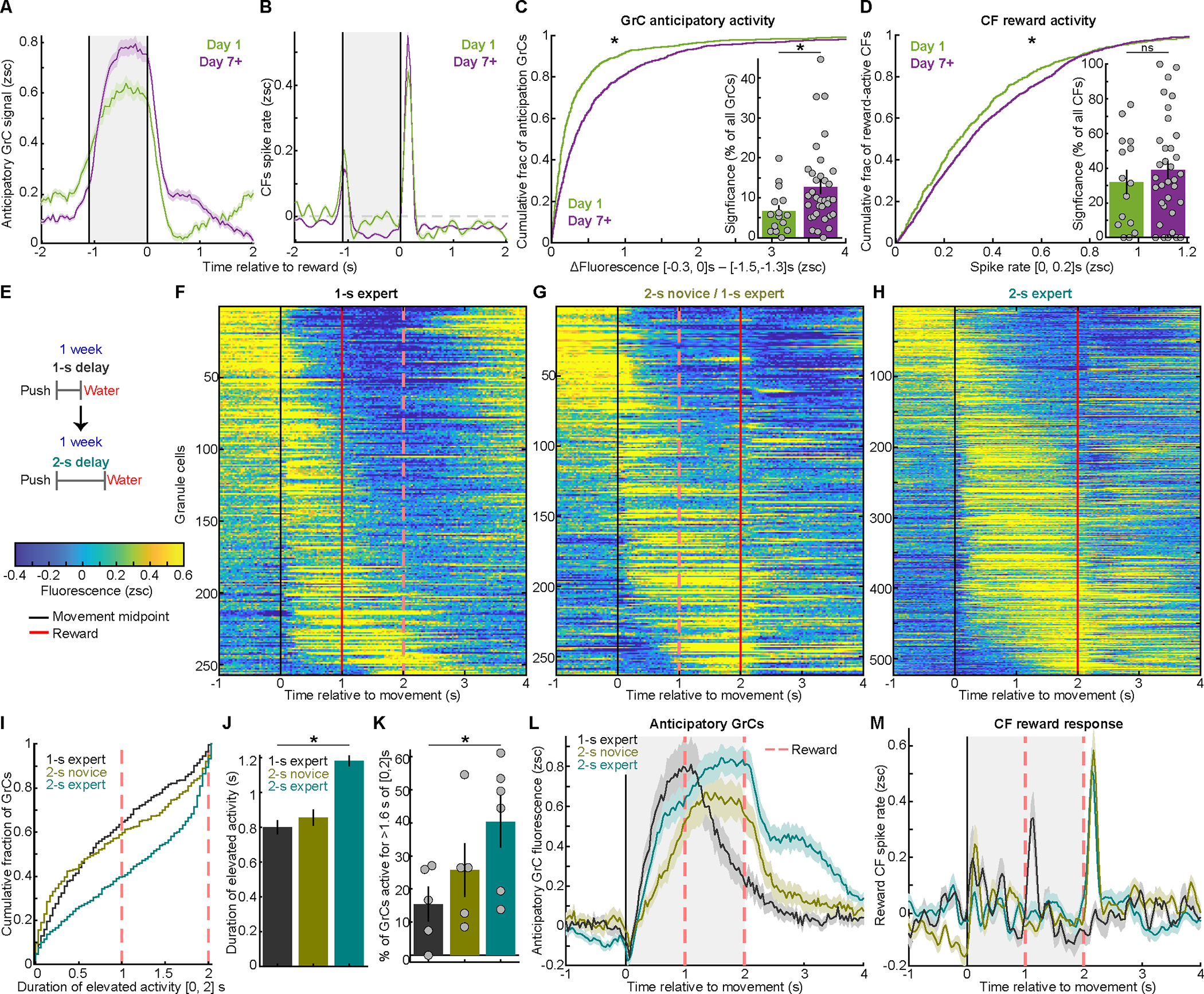
With behavioral learning, GrCs increasingly spanned the delay, while CFs persistently signaled reward **(A,B)** Average across GrCs with elevated delay-period activity (A, [−0.3 −0.03] s > [−1.5 −1.3] s and > [0.3 0.5] s), on Day 1 vs Day 7+ sessions. B, Average across CFs with elevated reward spiking (from [0 0.2] s). Cell/session/mouse counts: A-Day-1: 752/15/15; A-Day-7+: 1,135/33/19; B-Day-1: 657/15/15; B-Day-7+: 1,462/34/20. **(C,D)** With learning, GrC anticipation increased in magnitude (C, p<10^−6^, 752/1135 Day1/7+ GrCs), and in prevalence (p=0.02, 33/15 Day1/7+ sessions). CF reward responses increased in magnitude modestly (D, p=4×10^−4^, 657/1462 Day1/7+ CFs), and remained equally prevalent (p=0.56, 15/34 Day1/7+ sessions). Significance proportions: thresholded magnitude differences from A,B at 0.2 zsc and single-cell p<0.05; total #cells: 839/1964 Day 1/7+ CFs; 2,334/3965 Day 1/7+ GrCs). Dots show sessions. **(E)** A cohort of mice trained for a week on the 1-s delay task, before switching to a 2-s delay for another week. **(F-H)** 1-s-to-2-s retraining GrC responses in 5 mice. GrCs in each image are sorted by center of timepoints with elevated activity during [0, 2] s, relative to pre-movement levels [−0.8, −0.3] s. Cell/sessions counts: 257/5, 263/5, 523/6. 2-s-novice data averaged over first 50 trials to highlight earliest exposures; 1-s-expert and 2-s-expert averages over random 50-trial subsets. Color-bar applies to all panels. **(I,J)** Duration of GrC elevated activity between [0,2] s, relative to premovement levels [−1,0] s was significantly higher in 2-s-experts (p<10^−6^; 257, 263, 523 GrCs respectively). **(K)** The proportion of GrCs with elevated activity for >1.6 s during [0,2] s rose with learning (p=0.02, dots show 5/5/6 sessions respectively). **(L)** Average activity of reward anticipation GrCs (criteria: activity higher in final 0.3s before reward compared to early in the delay [0.1, 0.4] s; n=89/257, 105/263, and 247/523 GrCs per learning phase). Dashed pink lines denote 1-s or 2-s reward. **(M)** Average activity of reward-responding CFs (rate [0,0.2] s >0.1 zsc higher than [−0.3, −0.03] ms. 17/61, 34/71, and 35/143 CFs per condition).

To test whether GrC reward anticipation and CF reward spiking changed with learning, we imaged repeatedly as novice mice learned the 1.1-s delay (15 mice, 7.5±0.4 sessions per mouse, Figure S1D,E). We examined reward anticipation GrCs on Day 1 and Day 7+ (Figure 4A, spread across all Day-1 sessions/mice and 33/34 Day-7+ sessions from 19/20 mice). The magnitude of GrC anticipation grew and its prevalence increased moderately (Figure 4C). Thus, learning enhanced sustained GrC delay activity.

We next considered CF reward signals. In leading theories, CFs signal “errors” that drive GrC→PkC plasticity, which reduces future errors^60^. Both behavioral and GrC signatures of reward anticipation improved with learning (Figure 2A–C, 4A,C). Thus, if reward-evoked CF spikes signaled prediction errors, such spiking should decrease after learning. To quantify this, we identified CFs activated by reward (Figure 4B; spread across all Day 1 and Day 7+ sessions). We found that after learning, reward-evoked CF spiking marginally *increased* in magnitude and remained equally prevalent (Figure 4D), contrary to an error^61^. CF anticipatory suppression magnitudes and prevalence also increased (Figure S2Q). As an alternative to errors, CF reward spiking could mark the end of the expectation period spanned by anticipatory GrC activity.

### Learning lengthens GrC profiles to span the delay until reward

To directly test whether operant learning lengthened GrC profiles to span the delay, a cohort of mice trained with a 1-s delay for a week, and then retrained with a 2-s delay (Figure 4E). We examined GrC-CF profiles at three points: expert 1-s delay performance (Figure 4F); just after switching to the 2-s delay (Figure 4G); and expert 2-s delay performance (Figure 4H). In 1-s experts, most anticipatory GrCs were active mainly during [0, 1] s after forelimb movement, which was similar in 2-s-delay-novices (Figure 4I–L black/brown). After a week of 2-s training, elevated GrC activity largely spanned [0, 2] s following forelimb movement (Figure 4I–L turquoise; elevated activity sustained for ~50% longer, from 0.8±0.04s to 1.2±0.03s). Thus, learning lengthened the duration of many individual GrCs’ activity ramps. In addition, GrC differences between 1-s and 2-s delays were again inconsistent with licking motor signals *per se*: during [1,2] s after movement, when 1-s experts licked robustly to consume reward, 2-s-experts licked less, as they delayed anticipatory licking—opposite to the differences in GrC activation (Figure S3E–I). Consistent with 1s-delay data (Figure 4B), 2-s novices and experts both exhibited strong CF reward responses (Figure 4M). Thus, over days of training, GrC anticipatory profiles lengthened to span the delay between reaching and reward, which evoked persistent time-locked CF spiking. We hypothesized that lengthening GrC profiles to span the delay might help CF-guided GrC readout in PkCs.

### Learning increases reward timing information in GrC populations

**Figure 5 |:**
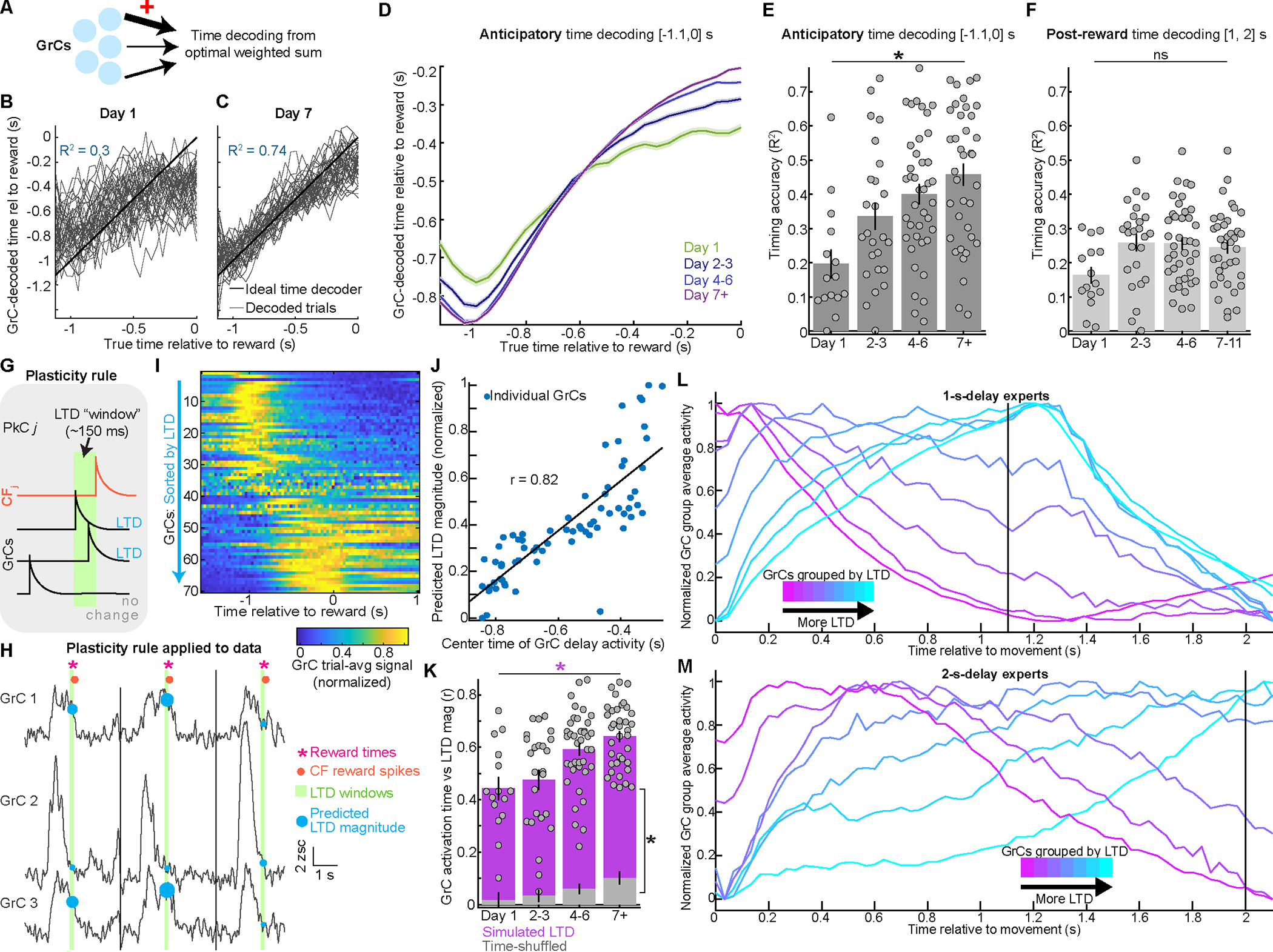
Learning yields GrC reward timing information that is computationally accessible to LTD **(A-C)** Linear regression (A, least squares, 10-fold cross-validated) to decode time to reward via weighted sum of GrCs ([−1.1, 0] s). Example decoding performance Day 1 (B) and Day 7 (C) (41 trials, 127 and 111 cells). **(D)** GrC time decoding output averaged across all trials (1,047, 2,242, 4,355, and 4,708 trials from 20 mice). **(E,F)** GrC delay time decoding accuracy (E) averaged across sessions (*p*=1.4×10^−5^. Dots show 15, 25, 40 and 37 sessions). Accuracy post-reward (F, [1,2] s) was persistently low (p=0.1) and substantially poorer than anticipatory decoding in experts (p<10^−6^). See also Figure S4A–D. **(G)** CF-dependent GrC→PkC plasticity rule. When a PkC (*“j*”) receives a CF spike, GrC inputs active in the prior ~150 ms are weakened (LTD, top two GrCs), but other GrC inputs are not (bottom GrC). **(H)** Simulating LTD on CF-GrC data. Example CF and GrCs in three trials centered on reward and concatenated (black lines denote trial breaks). Orange dots denote CF spikes within [0, 250] ms of reward. Each GrC’s activity in green LTD window ([-150, 25] ms from CF spike) was tabulated as predicted LTD between that GrC and the CF-recipient PkC. A logistic function bounded each LTD event between [0,1] ($\frac{1}{1+e^{-F/s}}$; F=avgGrCsignalinLTDwindow; ***s*=95**^**th**^ **percentile fluorescence per cell, Methods).** **(I)** Example session: GrC profiles sorted top to bottom by predicted LTD averaged over trials roughly ordered GrCs by time of peak activity during the delay (70 GrCs, 95 trials). Additional examples, Figure S4G–I. **(J)** For session in I, correlation between each GrC’s center of delay activity (x-axis, over [−1.1,0] s) vs its predicted LTD magnitude (y-axis; 70 GrCs, r=0.82, p<10^−6^, diagonal line, linear fit). Additional examples: Figure S4J–L. **(K)** Correlation from J across sessions grew with learning (magenta; p<10^−6^. Dots show 116 sessions; Days 7+ positive *r* at p<10^−6^, 37 expert sessions). LTD computed on time-shuffled GrC data had smaller correlations (p<10^−6^ 37 expert sessions). See also Figure S4O. **(L)** GrCs grouped by predicted LTD magnitude (percentiles computed for each session; percentile bin edges: [0,20,40,50,60,70,80,90,95,100]). Traces: average activity per GrC group (normalized to [0,1] to highlight differences in timing). Cell counts from bottom to top: 2077, 2068, 1025, 1057, 1032, 1036, 1038, 517, 522; 76 day-4+ sessions. See Figure S4M,N. **(M)** Same as L, for 2-s-experts (bin edges: [0,30,50,65,80,90,95,100]; counts: 157,103,80,78,53,26,26; 6 sessions).

After learning, many GrCs developed activity that spanned the delay, but for what computational purpose? Since animals successfully learned reward timing (Figure 2), we tested the quality of GrC reward time information via single-trial population linear time decoding (Figure 5A–C; 10-fold cross validated predictions). Learning substantially increased GrC delay time decoding (by 133%; Figure 5D,E). Did time decoding generically improve e.g., due to more consistent GrC activity? To test this, we decoded time *after* reward consumption and found persistently low accuracy (Figure 5F); by contrast, time decoding *during* reward consumption was already maximally accurate in novices (Figure S4A). Similarly, when comparing 1-s and 2-s paradigms, we found that 2-s expert GrCs were best able to decode 2-s of time passage, whereas 1-s decoding was more similar across conditions (Figure S4B–F). Thus, learning specifically enhanced GrC timing accuracy during the learned delay.

Learning generated new GrC temporal bases that enhanced population reward timing information. Prior data demonstrated that PkCs^62,63^ and cerebellar nuclei (CN) cells^64^ ramp in ways that may track delay periods. How might PkCs appropriately integrate numerous GrC inputs to readout their population timing signals?

### LTD simulated on anticipatory GrC bases predicts a gradient of GrC-PkC synaptic changes

Learning yielded GrCs with increasing reward timing information—could this information be extracted by PkCs? Among many other factors^65–68^, a major contributor to PkC output is integration of GrC inputs, partly directed by CF-driven synaptic plasticity. In classical cerebellar LTD, LTD weakens GrC→PkC synapses for GrCs that were active in the ~150 ms preceding a CF spike (for vermis^18^) (Figure 5G). We thus aimed to use the first simultaneous GrC-CF recordings to predict GrC→PkC LTD.

We recorded both the CF input and up to several hundred GrC inputs for several dozen PkCs. Therefore, for each PkC, we predicted LTD for each GrC input (simplifying by assuming that every GrC innervated every PkC). For each reward-evoked CF spike, we tabulated each GrC’s mean activity in the preceding plasticity window as a prediction of LTD (Figure 5H). We rectified these quantities to include only activity above baseline. We then computed the mean LTD for each GrC onto each PkC over all trials. To maintain overall PkC input strength, as likely achieved biologically by homeostatic^69^ and opposing^20,70^ processes like LTP, we normalized the range of this vector (**Methods**). Finally, we also computed the mean LTD across PkCs for each GrC, yielding a per-session prediction of GrC→PkC synaptic weights.

To visually compare GrC profiles to their predicted LTD, we sorted GrCs by LTD onto the average PkC. Surprisingly, this ordered GrCs by delay activity timing—even when peak activity long preceded the LTD window (Figure 5I, S4G–I). To quantify this effect, we described each GrC by the center of its delay activity. We scattered this “anticipatory center time” against predicted LTD (Figure 5J, S4J–L). The two quantities were correlated, and most strongly in experts—even for “center times” long preceding the LTD window (Figure 5K, S4O). As a control, this effect was absent when computing LTD using randomly reordered GrC traces (Figure 5K grey). Thus, reward LTD is sensitive to GrC timing across the preceding delay.

This result reflects specific GrC properties in our task: activity levels in the 150 ms LTD window near reward provided a powerful snapshot of GrC activity timing up to 2 s prior. To visualize, we examined activity of GrCs grouped by predicted LTD magnitude (Figure 5L, normalized in magnitude to highlight differences in timing). Indeed, progressively lower-LTD GrC groups exhibited progressively earlier delay activity ramps—even as ramps shifted hundreds of ms before the LTD window. However, these differences collapsed after reward.

While this strategy is mathematically effective for the 1.1-s delay, the basis in Figure 5L would be ineffective for 2-s delays. We thus tested generalization to a longer delay by computing LTD on our 2-s-delay expert data. This revealed a GrC basis analogous to that in 1.1-s-experts but “stretched” in time (Figure 5M, S4M,N). This demonstrates that learning a longer delay yielded a new GrC basis more suited to track the longer interval when guided by a similar but more temporally remote reward-evoked CF teaching signal.

Thus, we find that because learning yielded numerous GrCs that ramped activity up and down at different rates from forelimb movement until reward (Figure 3G), GrCs’ activity at reward provided a snapshot of their prior temporal profiles – even for GrCs that peaked up to 2-s earlier (Figure 5M, S4O). Thus, CF-driven GrC integration might enable PkCs to track delay passage.

### LTD from CF spiking at reward computationally suffices to readout GrC timing information

**Figure 6 |:**
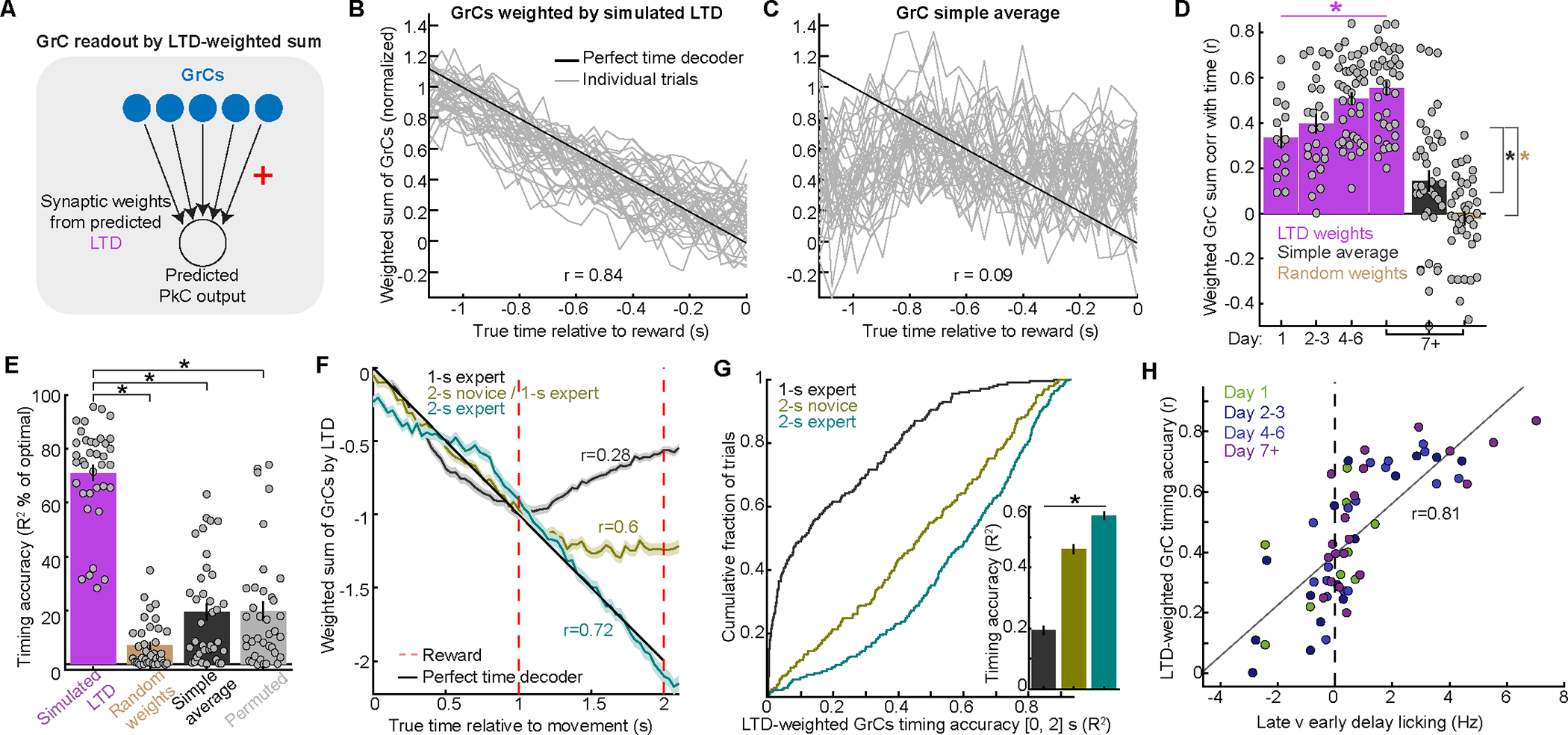
Simulated LTD-weighted GrC averages track time to reward for up to 2 seconds **(A-C)** GrC readout using LTD predictions of GrC→PkC weights (A). Example Day-7 single-trial LTD-weighted GrC sums (B) or simple GrC average (C). Correlation: time vs weighted sums (40 trials shown of 68 total, 111 GrCs). Normalization: range of trial-average scaled to [1.1,0]. Additional examples, Figure S5A–C. **(D)** Correlation between time and LTD-weighted GrC sums rose with learning (p=1.6×10^−5^, dots show 116 sessions from 20 mice). Simple GrC average or randomly reordered LTD-weighted sums poorly correlated with time (weaker than LTD-weighted, p<10^−6^). **(E)** In experts, LTD-weighted GrCs were far closer to optimal time-decoding than random weights, simple averages, or LTD computed on time-shuffled GrCs (* p<10^−6^, dots show 37 sessions). **(F)** For 1-s-to-2-s retraining data, LTD-weighted GrC sums using CF reward spikes (i.e., either 1-s- or 2-s-post-movement). Trial counts: 250, 250, 299; Mice/sessions: 5/5, 5/5, 5/6. **(G)** LTD-weighted GrC sum timing accuracy (R^2^) over [0, 2] s for data in F (p<10^−6^, 799 trials). **(H)** Session-by-session behavioral performance (late [-0.2, 0] s minus early [−0.8, −0.6] s delay licking) versus LTD-weighted GrC sum timing accuracy. Spearman r=0.81, p<10^−6^, 62 sessions. Diagonal line, linear fit.

If LTD is sensitive to GrC anticipatory timing, what computation could this facilitate in PkCs? We produced a minimal readout by summing GrCs weighted by the LTD weight vectors (Figure 6A), thereby predicting a possible component of PkC output (Figure 6B, S5A–C). The resulting GrC weighted sums correlated substantially with time through the delay, and increasingly so as learning progressed (Figure 6D, S5I,J). In experts, LTD-weighted GrC sums resembled optimal GrC time readouts in both accuracy and weights (Figure S5A–G,I–L). Thus, learning produced GrCs increasingly well-suited to LTD-based readout of delay passage until reward.

Given the prevalence and prominence of GrC anticipation signals in experts (Figure 3,4), might farsimpler GrC readouts suffice to extract timing information? We considered three trivial readouts: (1) simple average of all GrCs (Figure 6C); (2) GrCs weighted randomly (by reordering the above LTD weights); or (3) LTD simulated on time-shuffled GrCs. In each case, resulting GrC readouts only weakly correlated with time in experts (Figure 6D,E). Thus, even though learning enhanced GrC timing signals, this information could not be extracted trivially. Therefore, PkCs cannot “automatically” inherit timing signals, but setting synaptic strengths via LTD (with likely symmetric contributions from LTP, Figure S5K) sufficed to integrate GrCs into interval-tracking signals.

Are LTD-weighted GrC sums specific to the delay duration? We computed LTD on data from mice trained on a 1-s delay followed by retraining on a 2-s delay (Figure 6F). This yielded three results: (1) In 1-s-delay experts, GrC sums tracked time from [0, 1] s but the readout decayed from [1, 2] s; (2) In 2-s-delay novices, GrC sums tracked time from [0, 1] s but then *saturated* from [1, 2] s; (3) only in 2-s experts did GrC sums track time throughout [0, 2] s. Accuracy of 2-s-timing thus improved substantially (Figure 6G). Reward LTD can therefore extract at least 2 s of GrC timing signals–but only after learning appropriately lengthens GrC activity patterns to span the delay. Finally, to test whether readout quality related to behavior, we quantified lick timing specificity, which covaried with timing accuracy of LTD-weighted GrC sums (Figure 6H, S5H). Together, these data demonstrate that learning endows GrCs with anticipatory timing signals that could be integrated into a delay-tracking output via classical plasticity driven by reward-evoked CF spiking.

### PkC delay spiking ramps bear out predictions of LTD readout of GrC timing signals

**Figure 7 |:**
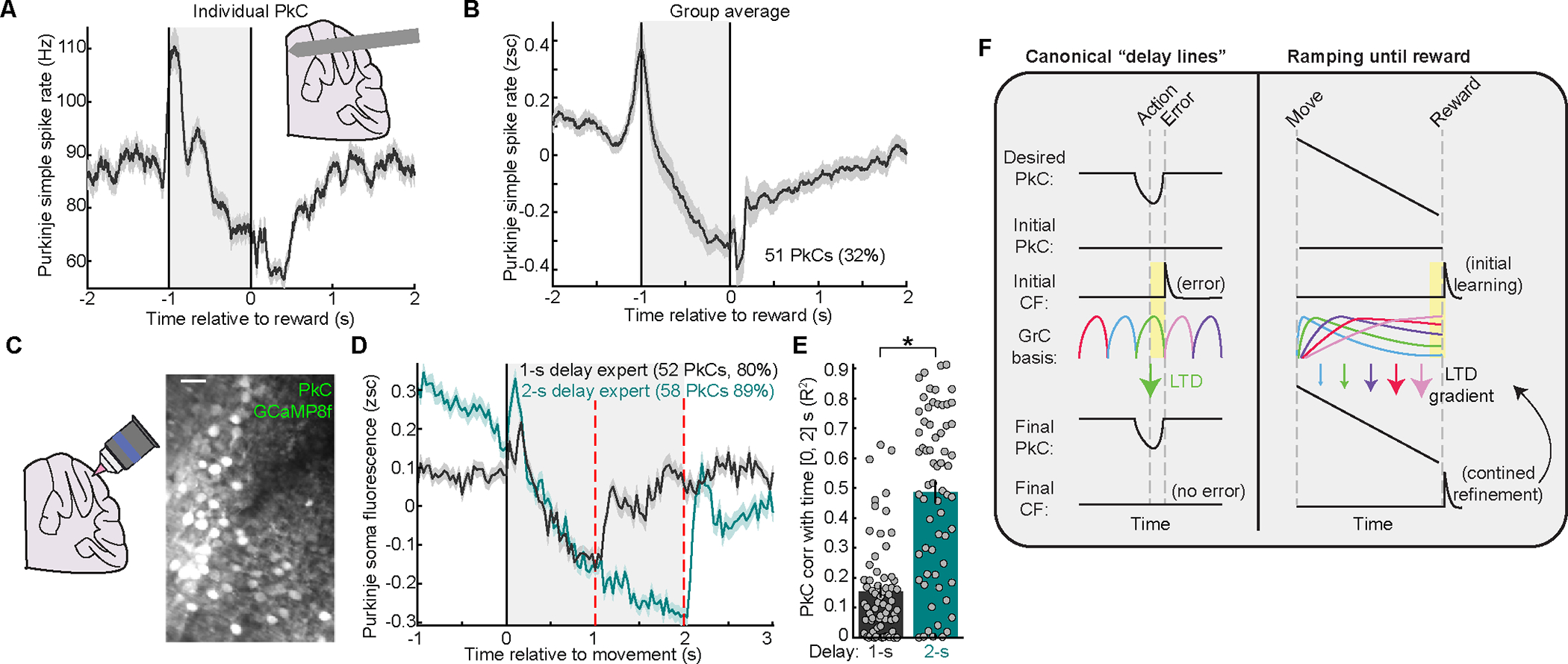
PkC simple spike ramps track interval from movement to expected reward for up to 2 s **(A-B)** Neuropixels PkC recordings. A, Example expert PkC simple spike (SS) rate ramped downward during the delay (124 trials). B, Average z-scored SS rate of PkCs whose delay SS rate decreased below baseline (negative slope [−1 0] s and negative zsc [−150 −25] ms; 32%, 51/162 PkCs in 4 mice/16 Day-7+ sessions; positive ramping cells in Figure S7). **(C-E)** Alternatively, we imaged PkC two-photon somatic Ca^2+^ in the region of our GrC-CF recordings, (C, mean *in vivo* image). D, trial-averaged fluorescence for PkCs with negative slope from [0, 1] s for 1-s-expert data (65/4/2 total PkCs/sessions/mice) or [0, 2] s for 2-s-expert data (65/3/2 total PkCs/sessions/mice). Timing accuracy [0, 2] s was higher in 2-s-expert PkCs (E, p<10^-6^). **(F)** Schematic. Left: A canonical strategy using a “delay line” GrC basis: erroneous actions trigger CF spiking. GrCs sequentially activate at distinct times for short durations. Learning adjusts synapses of GrCs temporally coincident with CF signal, eliminating both future error and CF error signal. Right: Strategy to produce PkC delay-tracking ramps from action to reward. CFs signal reward. During learning, GrCs profiles lengthen to densely span the delay with varying kinetics. Pre-reward LTD window provides snapshot of GrC ramping kinetics (like Figure 5L,M). Using classical LTD, CF reward spiking grades many GrC→PkC synapses by GrC anticipatory timing. LTD-weighted GrCs yield PkC spiking ramps from predictor to reward. Thus, new GrC basis sets that emerge with learning enable new types of PkC computation.

To test the prediction that a component of PkC simple spiking (SS) could track time to reward, we used Neuropixels (imec) to record PkCs in 1-s-delay experts (Figure S6A,B). We identified confirmed PkC SS units by their SS pauses after complex spikes (64 cells, Figure S6C–G) and putative PkCs based on their physical proximity (±100μm) to confirmed PkCs^71^ and similar spiking metrics (98 cells, Figure S6H–J,S7E). We computed mean SS rates aligned to reward in experts (Figure S7A). In both individual PkCs (Figure 7A) and among 32% of all PkCs (Figure 7B), SS rates gradually decreased with delay passage, as predicted (Figure 6B). Interestingly, an additional 25% of PkCs exhibited positive ramping SS, which tracked the delay with polarity opposite to our prediction (Figure S7B, Discussion). SS rates of many PkCs thus tracked the delay as predicted by readout of the GrC temporal basis.

Due to cerebellar and Neuropixels probe geometry, our electrophysiological recordings spanned multiple lobules and were more ventral than our dorsal-surface imaging sites (Figure S6A), such that few if any PkC recordings likely derived from the small region where we performed GrC-CF imaging. As an alternative, we used two-photon imaging for dense and restricted PkC recording in dorsal-surface right vermis Lobule VI. By monitoring Ca^2+^ in PkC somas with jGCaMP8f^72^, we obtained correlates of PkC SS rates^73^ (Figure 7C). Most PkCs exhibited downward delay ramping of somatic fluorescence (Figure 7D, S7F). Quantified by fluorescence correlation with time or its magnitude near reward, ~80% of PkCs negatively ramped with delay passage (Figure S7G,H), as predicted by GrC-CF signals in the same region.

To test the prediction that learning generates PkC ramps to span the learned delay duration (Figure 6F,G), we retrained PkC somatic imaging mice on a 2-s delay. As predicted and unlike 1-s experts, 2-s-expert PkC fluorescence progressively decreased through the 2 s delay, and returned to baseline after reward (Figure 7D green). Thus, only 2-s-expert PkCs conveyed 2 s of timing information (Figure 7E, S7I). The differences in PkC activity between 1-s vs 2-s experts were unlikely to be explained by licking, as PkCs exhibited large differences between 1-s and 2-s delays during [1, 2] s after movement that qualitatively conflicted with corresponding differences in licking (Figure S3F,G). Overall, PkC recordings bore out multiple predictions of LTD-based readout of delay-ramping GrCs. Since many PkCs tracked the delay until reward (Figure 7A–E), and since trivial GrC readouts cannot produce such signals (Figure 6D,E), it is thus likely that GrC LTD and/or computationally similar mechanisms like LTP (Figure S5K) contribute to delay-tracking PkC SS ramps.

## DISCUSSION

Using simultaneous GrC-CF imaging during operant conditioning for delayed reward, we found that learning yielded numerous GrCs that ramped activity for up to 2 s from forelimb movement until reward; reward then triggered widespread time-locked CF spiking (Figure 3,4). Via simulation, we found that canonical LTD applied to this GrC temporal basis could grade GrC→PkC synaptic weights by GrC anticipatory timing (Figure 5). We thus predicted and confirmed that many PkCs could generate interval-tracking SS ramps from movement until reward (Figure 6,7). Newly-learned GrC bases were also specific to behavioral timescale: GrCs in 1-s-experts were ill-suited to 2-s interval tracking, but 2-s-delay retraining “stretched” GrC profiles to span the longer delay (Figure 4,5,S4). This process mirrored behavioral learning (Figure 2,S2) and accounted for PkC ramp differences between 1-s and 2-s-delays (Figure 7,S7). Given the behavioral importance of long interval-tracking, ubiquitous neural ramping in the forebrain and its importance for perceived time passage^74,75^, and recent findings of cerebellar PkC and CN neural ramps^62–64,76,77^, these GrC temporal bases and their interplay with CF reward signals may be common elements of cerebellar computation.

### Learning yields GrCs that link predictors to temporally remote CF reward signals

Decades of theory posited GrC representations of time^28–36^ such as the “delay line” GrC basis^26^ (Figure 7F left). Contrastingly, in our expert data, up to 40% of GrCs simultaneously activated for up to 2 s until reward (Figure 7F, right). Because different anticipatory GrCs activated with different kinetics, each GrC’s activity level just before reward provided a snapshot of its prior temporal profile (Figure 5L,M). Rather than redundancy, “dense” GrC activity more finely sampled the delay. By sustaining activity from movement until reward, GrCs that activated early were “linked” to reward-evoked CF spiking much later. Thus, GrCs in our data are well-suited to generate *interval-spanning ramps via a remote CF instructive signal*, whereas a delay line basis will generally not (Figure 7F left, Figure S5M–P). Since these GrC bases scaled temporally across delay durations (Figure 4), it is possible this basis might also “compress” to shorter durations, and thus help drive anticipatory PkC suppression in classical cerebellar short-latency associative learning^78,79^.

Our central finding is that learning yielded new GrC activity profiles that rose and fell over seconds to link a predictor to temporally remote reward-evoked CF teaching signals. This claim is agnostic to the mechanism for GrC alterations. Nevertheless, sustained activity ramps often reflect recurrent excitatory circuits^80,81^, as found in the cortico-cerebellar loop and the nucleocortical loop, which exhibits structural plasticity that could help amplify specific mossy fiber input patterns to GrCs^82^.

### Persistent CF reward signals

Canonically, CFs were thought to signal “errors,” such that learning eliminates both error and CF error signal. Often, CF representations are more complex^61,83–86^. Here, reward-evoked CF spiking persisted throughout learning, contrary to an error (Figure 4). We posit two possible explanations for this result. First, expert CF spiking could help transition or initiate subsequent behavioral states, in this case from reward anticipation to consumption. Intriguingly, other studies have also reported behaviorally time-locked CF spiking that either emerged, solidified, or persisted during learning, with stronger expert CF spiking sometimes associated with better performance^44,60,79,83^. Second, expert CF reward spiking could help to continually refine GrC integration. In contrast to a sparse GrC basis where persistent CFs might excessively weaken target GrCs relative to those active just outside the LTD window (Figure 7F left), we predict that CF reward spiking grades many GrC→PkC synapses at once (Figure 7F, right). If paired with homeostatic or opposing mechanisms^20,69^ to maintain overall PkC input strength, this strategy could yield stable synaptic weights and learning.

### Impact of delay-spanning GrCs on PkCs

Recent studies found extended ramping in PkCs^62,63,76^ and CN^64,77^ neurons, raising the question whether ramps were “inherited” from upstream or could result from cerebellar computation. PkCs in our task also ramped over seconds-long delays (Figure 7, S7). But our GrC-CF recordings permitted two key discoveries: (1) *interval-tracking PkCs cannot be generated by trivial GrC readout* (Figure 6C–E), despite widespread sustained GrC activity; but (2) reward LTD *computationally sufficed* to integrate diverse GrCs into seconds-long interval-tracking PkC ramps (Figure 6F). We thus posit that learning involves *both* progressive changes to GrC activity *and* changes in its readout in PkCs. An open question is whether these changes occur together or sequentially (Figure S5H).

A limitation of our technique is that we sampled GrC inputs physically closest to CF-recipient PkCs, but GrCs contribute from distances up to ~1 mm^87^. Though GrC profiles in our task varied minimally at this spatial scale^9,41^, we cannot exclude differential roles for distant GrC inputs. Furthermore, our simulation assumed the classical plasticity rule in which CF reward spikes lead to GrC→PkC LTD, but our data does not provide direct evidence for CF reward spike LTD. Nonetheless, while LTD is modulated by factors unobserved in the present study, it is generally thought that most CF spikes lead to some LTD^88^. In addition, while we predicted GrC→PkC connection strengths, no technique can yet quantify connection strengths among hundreds of cells registered to their activity during learning. Instead, we tested and confirmed predictions about PkC ramping. While many PkCs bore out predicted delay ramps, in our electrophysiological recordings some also ramped upwards*—*unexplainable by LTD alone, but consistent with prior findings of bidirectional PkC modulation^22^. Such PkC features could reflect numerous synergistic mechanisms: the balance of LTD versus LTP (triggered by reduced CF spiking^20–22^; Figure S5K); interplay of GrCs and interneurons, which could contribute to PkC ramping by driving SS below baseline (Figure 7); lasting rises in PkC Ca^2+89^; and changes in PkC excitability^69^. On the other hand, upward-ramping PkCs were rare when imaging near the site of our GrC-CF recordings (Figure 7D,E, S7F–I). Functionally, all ramps may help track intervals between events.

### Function of ramping GrC bases and delay-tracking PkCs

The cerebellum has long been thought to synchronize actions to a short latency prior to an event^90^ (Figure 7F, left). Increasing data suggests that the cerebellum may also contribute to continuous timing functions where ramping signals predominate^62–64,76,77^. In our data, cerebellar delay ramping resembled a temporally building expectation of reward (Figure S3). Nevertheless, our data cannot adjudicate whether the cerebellum generates such ramps (a) solely when needed to drive temporally building licking when awaiting reward; or (b) also to help maintain internal estimates of time passage generally. We favor (b) because: PkCs ramp in differing behavioral contexts and cerebellar regions^64,77,91^; cerebellar ramps are tightly linked to those in neocortex^9,64,91^, which have generalized temporal expectancy functions^92–94^; and the diversity of CN targets^95,96^ implies broader utility than driving a fixed set of motor effectors.

How might such signals influence behavior? Activating PkCs during the delay transiently terminated licking, potentially by inhibiting CN cells projecting to areas driving motor output. The transient manipulation also disrupted subsequent lick timing relative to expected reward (Figure 2J, S2J), possibly by disturbing internal estimates of time passage. Finally, repeatedly perturbing PkC activity prevented the relearning of new reward timing even when probed on laser-off trials (Figure 2M), possibly by disrupting signals used for temporal learning. However, it is important to note that this perturbation disrupted spontaneous PkC spiking in addition to GrC-driven spiking, leaving the possibility that direct GrC perturbation might yield a different constellation of effects.

Long-interval time-tracking via ramping signals is supported by decades of forebrain studies indicating its importance for both estimation and perception of time in multiple species^74,75^. For the cerebellum, we demonstrate that learning leads to new sustained GrC bases that link a predictor to distant reward-evoked CF teaching signals—a strategy well-suited to learn to track long time intervals. Cerebellar learning of interval-tracking ramps may characterize its interactions with the forebrain and contributions in cognitive domains generally.

## STAR★METHODS

### Resource availability

#### Lead contact

Further information and requests for resources and reagents should be directed to and will be fulfilled by the lead contact, Mark Wagner (mark.wagner@nih.gov).

#### Materials availability

This study did not generate new unique reagents.

#### Data and code availability

Data will be deposited at Dryad at the time of publication.

Source code will be deposited at GitHub at the time of publication

Any additional information required to reanalyze the data reported in this paper is available from the lead contact upon request.

### Experimental model details

#### Mice

All GrC-CF imaging experiments used Math1-Cre / Ai93 (TIGRE-LSL-TRE-GCaMP6f) / ztTA (R26-CAG-LSL-tTA) mice, aged 6 – 16 weeks, which, in the cerebellum, express GCaMP6f selectively in GrCs^41^. Optogenetics experiments used double transgenic crosses of PCP2-Cre^97^ mice to Ai32 mice^98^ (Jackson labs), while electrophysiology and PkC somatic imaging used WT mice. All procedures were approved by both the NIH and Stanford animal care and use committees.

### Method details

#### Virus

An abridged PkC-specific promoter (L7–6)^52^ drove expression of the red Ca^2+^ indicator R-CaMP2^51^, packaged into AAV–L7–6–R-CaMP2. PkC imaging used AAV-ef1A-jGCaMP8f virus. Virus injected at ~10^12^ genomes/mL.

#### Viral injection

Under isoflurane anesthesia (~2% in ~1L/min of O_2_), we cleaned the scalp and removed hair. We made a sagittal incision in the skin to expose the underlying skull, which we then lightly scraped free of soft tissue. We drilled a ~300 μm hole over the vermis of Lobule VI (~0 to 0.3 mm right of midline, on the post-lambda suture). We inserted a ~25 μm diameter-tip glass capillary ~300 μm below the pial surface and injected 500 nL of AAV-L7–6-R-CaMP2 (or jGCaMP8f for PkC surgeries) into the tissue and waited 5 minutes after injection before withdrawing the tip. Virus was typically allowed to express for 10 to 14 days prior to imaging.

#### Histology

Mice were deeply anesthetized via IP injection of 5% tribromoethanol in PBS, and then transcardially perfused with PBS followed by 10% formalin, after which the brain was removed and left overnight in 10% formalin. We cut 60 μm sagittal sections from the cerebellum using a vibratome (Leica VT 1000S). The sections were then washed in PBS.

For GrC-CF histology, we performed immunostaining first by 1h of blocking in 10% FBS in PBST. We dual-stained the sections using chicken anti-GFP (Aves Labs, GFP-1010) and rabbit anti-RFP antibodies (AB-62341) for ~48h, followed by a 0.1% PBST wash, followed by Alexa 488 anti-chicken (A-11039) and Alexa 594 anti-rabbit (A-32754) secondary antibody stainings for ~3 h. After mounting the sections, we imaged the resulting slides using a confocal microscope with 488 nm and 561 nm lasers (Zeiss LSM 510).

For PkC Neuropixels histology, we directly mounted the sagittal sections in DAPI mounting medium and imaged using the 405nm and 531nm lasers.

#### Window and headplate implantation

Implantation followed viral injection. We removed a patch of skin with a mediolateral span from ear to ear, and a rostrocaudal span from the back of the ears to the eyes. We scraped the entire exposed skull surface free of soft tissue, and then used VetBond (3M) adhesive to seal the edge of the skin incision to the skull. We drilled a ~3.5 to 4 mm diameter cranial window centered over the right vermis of lobule VI. We then affixed a #0 or #1 3 mm diameter glass cover slip onto a 3 mm outer diameter, 1 mm height, 2.7 mm inner diameter steel ring, using UV curing optical adhesive (Thorlabs NOA 81). We stereotaxically inserted the glass-ring combination into the cranial window at a 20°–25° angle counter-clockwise from the sagittal axis and a 40°–45° degree azimuthal angle from the vertical axis. We depressed the glass to a depth below the average depth of the underside of the skull at the window perimeter. We then sealed the outer face of the steel ring to the skull using Metabond (Parkell).

We then implanted a custom headplate with a 5 mm central opening and two extensions with two holes for screws to affix the headplate to fixation bars. We stereotaxically lowered the headplate, surface parallel to the glass window, onto the skull with the central opening centered on the window, and then covered the entire exposed skull with Metabond up to the top surface of the headplate.

#### Behavioral data collection

As described previously^56^, our operant device was a two-axis robotic manipulandum consisting of two DC motors, two high-resolution optical rotary encoders, and a 4-linkage two-degree-of-freedom manipulandum configuration. The manipulandum was controlled via a custom programmed nested series of feedback loops across an FPGA, PC with real-time Linux OS, and Windows PC (together forming the National Instruments cRIO platform). In addition to handling real-time control tasks, the apparatus recorded the x and y position of the handle of the manipulandum at 200 Hz. We also synchronously acquired at 200 Hz: a readout of the solenoid trigger that released water reward; the frame counter from the two-photon microscope; in some cases, a capacitive lick sensor reading; and in some cases, the optogenetic laser trigger pulses. Reward delivery was triggered either via windows PC (the main GrC-CF 1.1-s-delay dataset), or directly in the Real-Time OS (all other datasets: 1-s-to-2-s learning data; PCP2-Cre/Ai32 ChR2 data; Neuropixels recordings; PkC somatic imaging). The latter eliminated roughly ~100 ms of delay variability; the resulting data was qualitatively similar between methodologies.

In addition, in some cases, we collected behavioral video data at 30 – 120 Hz (Imaging Source). This was synchronized to the microscopy data either by hardware frame trigger or by automatically identifying the frames on which the IR laser shutters opened and closed. Finally, we used DeepLabCut^99^ to semi-automatically annotate 2D locations of the mouse’s two forepaws, nose, tongue, eye pupil, or whiskers.

For some mice, we obtained capacitive sensor lick readings (26 mice total; main dataset: 12 mice; PCP2-Cre/Ai32 ChR2 dataset: 7 mice; 1-s-vs-2-s imaging dataset: 5 mice; PkC somatic Ca^2+^ imaging dataset: 2 mice). Capacitance measurements were sensitive to gross changes in positioning or degree of contact, such that the sensor sometimes became ‘stuck’ in the ‘on’ state. We automatically excluded from licking analysis any trials in which the sensor was “high” for more than 90% of any continuous 1.5-s block of time. For the remaining trials, we computed lick rate by identifying lick onset events in the raw binary sensor contact traces. Finally, we filtered the binary traces to produce smoothly-varying rate signals.

#### Behavioral training

During active behavioral training, mice were water restricted to 1 mL per day, weighed daily for excess loss, and monitored for signs of lethargy, coat deterioration, hunching, and general distress. Mice obtained water until satiety during behavioral training and received remaining water up to the 1 mL dosage in the home cage.

Reaching tasks employed our custom two axis robotic manipulandum^56^. The linear reaching task had the following structure. Mice self-initiated trials by pushing the handle of the robotic arm. Trials were terminated when either of two conditions were met: (1) animals reached the 8 mm virtual “wall” where the robot immediately terminated movement; or (2) animals ceased pushing the handle at any distance >3 mm for more than 100 ms. Following a delay after the end of a successful (>6–7 mm) movement, the computer dispensed (via a gravity-fed reservoir through a solenoid valve) a water droplet from a gavage needle in front of the animal’s mouth. The main GrC-CF imaging dataset had a 1.1-s-delay; for all other datasets including optogenetics, 1-s-to-2-s behavioral and imaging studies, Neuropixels recordings, and PkC somatic imaging, delays were either 1-s or 2-s as indicated. Following an additional ~2 s delay, the robotic arm automatically returned to the animal to self-initiate the subsequent trial.

Mice underwent typically 1 to 3 days of pretraining during which we did not perform imaging, until animals reached ~50 trials in 30 minutes. The function of pretraining was to minimize sessions in which animal brains were exposed to laser light without producing any behavioral imaging data. After this, we imaged during all subsequent training sessions for ~1 week, returning to the same field of view each day. After the week of chronic imaging, we sometimes imaged distinct imaging fields for 1 to 3 additional days.

#### Two-photon microscopy

We used a custom two-photon microscope with a resonant-galvo x-y scanhead (Cambridge Technology. Galvo, 6215H; resonant, either CRS 8k or CRS 12k). The brain was illuminated by two lasers, one at 920 nm for GCaMP6f (typical power: ~60 mW), and one at 1064 nm for R-CaMP2 (typical power: ~40 mW). In some cases, 920 nm was provided by a fixed wavelength 920 nm laser (Spark Alcor 920 2W), while in other cases it was provided by a tunable Ti:Sapph (Spectra Physics MaiTai). 1064 nm illumination was provided by a fixed wavelength laser (Spark Alcor 1064 2W). The 1064 nm laser path passed through an electrically tunable lens (Optotune, either EL-10–30-CI-NIR-LD-MV or EL-16–40-CI-NIR-LD-MV), in some cases with an additional offset lens (75 mm, 125 mm, or 150 mm focal length). The emission path split red and green onto two PMTs.

The main dataset was acquired using a 40x magnification 0.8 numerical aperture (NA) objective (Olympus XLUMPlan). The image size was 512 × 512 pixels over a field of view of 234 × 234 μm. In some cases (those using the CRS 12kHz scanner), the 1064 nm (red) and 920 nm (green) data were acquired via alternating frames by alternately shuttering the two lasers, resulting in an effective frame rate of 22.5 Hz. In other cases (those using the CRS 8 kHz), the two channels were acquired simultaneously at 30 Hz.

To select a field of view, we began by locating the region of cerebellum with viral expression of R-CaMP2 in PkCs. From this restricted region, we then optimized for the region with the best joint SNR in both GrCs and PkCs. We then optimized GrC imaging depth using the objective z-piezo, and finally optimized the PkC dendrite imaging depth using the Optotune remote focusing adjustment.

In cases of chronic imaging, following the first day of two-photon imaging, we used the wide field camera image to move to the nearest major vasculature landmark, collected an image of the landmark, and recorded the distance from our chosen imaging field to the landmark via the X-Y-Z sample translation stage distances. To return to the same imaging field on subsequent days, we inverted this procedure by finding the landmark and moving the recorded distances to the imaging field. We also recorded the final Optotune remote focusing offset for the 1064 nm laser. We then fine-tuned the GrC imaging field by comparing the image to the mean image from the first day of chronic imaging, ensuring that landmark GrCs from the initial session were present in the daily field at several locations in the imaging frame.

#### PkC somatic Ca^2+^ imaging and analysis using jGCaMP8f

We followed the surgical preparation described for GrC-CF imaging, with the only difference being the injection of AAV1-EF1A-jGCaMP8f. After 10 days, we examined the mice under the two-photon using a Nikon 16x 0.8 NA objective, and localized the PkC layer, picking imaging fields with the greatest number of infected PkC bodies.

#### Optogenetic studies

We crossed PCP2-Cre^97^ mice to Ai32 mice^98^ to generate PCP2/Ai32 double transgenics and implanted cerebellar windows as described above. After a week of training, we acquired perturbation data in expert mice. We positioned a ferrule-terminated multimode optical fiber (200um core, 0.39 NA, Thorlabs MR83L01) ~1 mm above the glass centered on Lobule VI. A 488 nm laser (Coherent OBIS LX) delivered 5–15 mW (measured in CW at fiber tip) via a Fiberport (Thorlabs PAF2–7A). On interleaved laser-on trials, the robotic controller waited for the mouse’s voluntary movement initiation and, when the handle crossed a 6–7 mm distance threshold, the FPGA waited a fixed period before delivering a fixed number of 5-ms TTL pulses with 15 ms interval to the OBIS modulation input. The following table summarizes parameters for each experiment: Experiment As we did not mask the blue light, for blue light controls, we used opsin-negative transgenic imaging mice with cerebellar windows and performed the same procedure. For LIX controls, we exposed LIX beneath the soft tissue in the neck and positioned the fiber behind the cerebellum.

**Table:** 

Experiment	Laser-on (mean %)	Laser-on, mean % of rewarded	Laser-on, % of omitted	Laser-on time (s from reward)	Laser-off time (s from reward)	Number of pulses
Figure 2I,J	22	17	50	−0.83±0.006	−0.05±0.006	36±0.3
Figure S2J	10	0	50	−0.94±0.004	−0.58±0.003	15±0.06
Figure S2K	34	31	50	−1.1±0.01	0.88±0.02	61±0.4
Figure S2L	17	8	50	−0.8±0.002	−0.02±0.002	32
Figure 2M,N	90	100	0	1.66±0.003 (from reach)	2.25±0.003 (from reach)	25

#### Neuropixels recordings

Under anesthesia as above, mice were implanted with a headplate. Mice were water restricted and trained for 1 to 2 days before initial recordings. The day before recording, we opened a <1 mm craniotomy in the same location used for imaging, and then covered the hole with Kwik-Cast (WPI). The following day, immediately before recording, we pierced a small hole in the dura with a needle. We then inserted the Neuropixels probe stereotaxically and under visual control, directly through lobule VI from behind the animal, using an azimuthal angular range of 79–84 degrees, and a mediolateral angular range of 0–15 degrees. We varied the angle across recording days to avoid piercing the same tissue multiple times. We inserted roughly 2 mm, to reach the anterior end of the cerebellum, and thus passing primarily through Lobule VI and V. After confirming likely passage through multiple PkC layers as viewed in real-time in spikeGLX, we allowed the tissue to settle for 5 to 10 minutes, before initiating a recording session during behavioral training for 18 minutes. We programmed our NI cRIO behavioral apparatus to output synchronization pulses to the Neuropixels NI DAQ. For the final two recording sessions for each animal, we coated the electrode in DiD for histological track visualization.

### Quantification and statistical analysis

#### Image processing

Green and red channels were independently motion-corrected using a sequence of rigid followed by nonrigid NORMCORRE registration^100^, as we empirically found that brain motion at the molecular layer differed from that at the GrC layer. To correct for slow, full-frame changes in fluorescence that did not correspond to cellular activity, we fit a double exponential to the frame-averaged fluorescence across the entire movie. For each frame, we then divided every pixel by the exponential fit value for that frame, and then manually confirmed that the resulting frame-averaged fluorescence trace was roughly flat across the recording.

#### Cell identification and signal preprocessing

To identify the spatial locations of GrCs, we used a pipeline based on cNMF^101^. We initially used cNMF to identify candidate GrCs. We automatically discarded candidates whose central regions were too small (<~0.0005mm^2^) or too large (>~0.003mm^2^). We then manually discarded candidates in cases where either (1) the spatial filter clearly did not correspond to GrC somas, or (2) where no clear elevation of fluorescence was observed in the video at the spatial location of the cell filter during high points in the cNMF signal trace. We then manually annotated the movie for “missed” cells and “seeded” a second round of cNMF with these additional candidates and then repeated the above steps.

To identify the spatial locations of PkC dendrites, we used a pipeline based on PCA/ICA^53^. We again automatically discarded candidates based on size as above.

In some cases, we then applied a convolutional neural network classifier to automatically discard additional candidates whose spatial filters clearly did not correspond to Purkinje dendrites We built our neural network with 15 layers: one image input layer, two repeats of a convolution-batch normalization-ReLU-average pooling layers sequence, a convolution-batch normalization-ReLU layers sequence, and a fully connected-soft max-classification layers sequence. We trained the classifier over eight epochs and shuffled the training data before each. We used an L2 regularization factor of 0.01, an initial learning rate of 0.001 with a piecewise learning rate schedule, and a mini batch size of 32. We trained this neural network on 7,400 previously sorted PkC dendrite spatial filters from five mice, across 74 sessions. The training set consisted of 4,213 (56.93%) images that visibly contained PkC dendrite spatial filters (class 1) and 3,187 (43.07%) images that did not (class 0). The final neural network had an accuracy of about 90%. We then discarded the candidates that were not classified as PkC dendrite spatial filters and manually inspected those remaining. In addition, we manually split spatial filters that clearly contained more than one PkC dendrite. Finally, we automatically searched for possible duplicate dendrites or candidate pieces of split dendrites to be merged, and manually removed identified duplicates and manually merged identified split dendrites.

For both GrCs and PkC dendrites, we then back applied the final set of spatial filters to the preprocessed movies, to yield the initial cell activity traces. For each cell, we next removed signal drifts on timescales slower than neural activity by subtracting off a 10^th^ percentile filtered version of the signal (GrCs: 10 s moving window; PkC dendrites: 5 s).

Finally, we uniformly scaled the signal magnitudes across cells by normalizing the amplitude of their baseline (noise) fluctuations (initial signal magnitudes were ambiguous due to: lack of meaningful physical units; differences between cells in SNR & indicator expression levels; changes in SNR or brightness within a session; ambiguous impact of spatial filter pixel weighting). For each neuron, we first estimated the center of the noise distribution, by computing a (slow) moving median (GrCs: 2-minute window; PkC dendrites: 1-minute window), but we excluded any timepoints above the cell’s 99^th^ percentile value, which were Ca^2+^ transients. For each cell, we subtracted off this estimate to center the noise distributions on zero. Next, we determined the noise fluctuation magnitude, by computing a slow moving windowed standard deviation on all fluorescence values below zero (excluding the top half of the distribution removes neural activation transients). This corresponded to the lower half-normal distribution of fluorescence noise fluctuations. We divided each cell’s trace by the estimated noise standard deviation $\sigma \sqrt{1-\frac{2}{\pi }}$. Overall, this produced signals where zero was defined as the center of the noise distribution, and the noise standard deviation was normalized across cells to unity magnitude, i.e., z-scored.

Since PkC dendritic calcium transients reflect complex spikes^44^ and thus individual CF spikes, we also estimated the time of individual CF spikes. We deconvolved out the indicator kinetics (*τ* = 150 ms) from the activity trace and thresholded the resulting signal (1.9 s.d.) to identify individual transient times. When further producing single-trial spike rates, we filtered the binary event traces with a 200 ms kernel.

#### PkC somatic Ca^2+^ imaging analysis

We again used cNMF to identify active PkC bodies. During manual curation, we accepted candidates that, in addition to having the expected size and shape, clearly exhibited elevated fluorescence in the z-scored movie during their nominally “brightest” frames. Finally, since it was typically impossible to restrict the entire imaging field to the exact depth of the PkC layer, we often observed some contamination from PkC dendritic signals (complex spikes). We therefore manually excluded any detected PkC somas that exhibited many fast transients with the visual appearance of dendritic complex spikes (i.e., a single transient that decays in ~200 ms), as these were excessively contaminated by dendritic fluorescence. The resulting remaining signals were passed through the same postprocessing pipeline described in the previous section for GrCs.

#### Neural response analysis

For trial-aligned analyses, we began by identifying all reaching movements from the manipulandum position data. To align trials, we sequentially identified: the time of reward delivery; reach “end” and “midpoint” as the times when the y position first extended to within 0.5 mm of its maximal value or beyond 4 mm respectively; reach “start” as the time when a (100 ms-window-smoothed) velocity estimate fell to below 15 mm / s, prior to the midpoint. To restrict analysis to stereotyped trials, we identified reaches >7 mm in length. When analysis of the main dataset required simultaneous alignment to reward and reaching, we concatenated the reach-aligned and reward-aligned neural data such that movement midpoint uniformly occurred at −1.1 s relative to reward.

#### Neural learning analysis

Our main dataset consisted of 20 mice. Of these, in 15 we imaged across multiple days of learning, aiming for 7 days of repeated imaging of the same neurons, followed by an additional 1 to 3 days of imaging distinct fields of cells to sample more neurons. On average we obtained 7.2+/−0.1 repeated imaging days in these 15 mice. Across all 20 mice, we obtained expert imaging data from, on average, 1.7+/−0.1 distinct fields of view.

For analyses in which the relevant N values were unique cells (e.g., Figure 3), all included sessions were taken from distinct fields of view (no repeats).

For analyses in which the relevant N values were unique sessions (e.g., Figure 5D–F), we included all sessions.

#### Temporal decoding analysis

We used a 10-fold cross-validated linear regression (ordinary least squares) analysis to determine the accuracy of GrC temporal decoding. For each session, we first produced a matrix of size N_GrC_-by-T-by-N_T_, where N_GrC_ was the number of GrCs, T was the number of imaging frames corresponding to 1.1 second, and N_T_ was the number of rewarded trials. We concatenated all trials along the time axis to produce a matrix of size (N_T_ x T)-by-N_GrC_. We then produced a “target” temporal axis vector of length T, whose entries ran from -1.1 s to 0 s (to span the delay), and then repeated this vector N_T_ times to produce a target vector of size (TxN_T_)-by-1. From the cross-validated linear regression results, we retained the cross-validated prediction of the temporal axis for each trial and computed the resulting accuracy (R^2^) and mean absolute error in ms. For post-reward temporal decoding, we repeated the identical procedure on 1 s of post-reward data and a time axis spanning 0 to 1 s.

#### Neuropixels analysis

We used Kilosort 2.5 to perform spike sorting. For all recordings (27 sessions across 4 mice), we applied the common average reference filter to the raw data prior to running Kilosort^102^ and used the following parameters: threshold = [10 3], high pass filter = 100 Hz, lowpass filter = 5000 Hz. To visualize and perform quality control on all of the clusters, we used the Phy^103^ template GUI and the ecephys^104^ quality metrics module.

Initially, we eliminated clusters with less than 100 spikes. We calculated the firing rate for each cluster in 30 second windows and discarded those that fell outside the appropriate ranges (40 Hz-200 Hz for simple spikes, 0.4 Hz-3 Hz for complex spikes).

We confirmed PkC identities based on cross-correlation analysis to putative complex spike clusters, with a characteristic SS “pause” of ~5–20ms after a CS (Figure S6). We then used confirmed PkCs as estimates of the physical location of PkC layers along the Neuropixels shank (±10 channels/100μm^71^). Clusters within each PkC layer were considered potential simple spike clusters based on minimum spike rate (40 Hz), inter-spike interval violations ratio (<2%), waveforms, and oscillatory auto-correlograms.

To handle cells that were not present for the entire recording session, we computed the spike rate per trial, and then the 75^th^ percentile value across all trials with a mean rate >5 Hz, and defined a presence threshold as half this value. We then found the first and last trial that exceeded this threshold. Across 162 putative PkCs, by this metric the average cell was present for 13±0.3 min, or 81±2% of the total recording length.

#### Statistics

A detailed panel-by-panel summary of all statistical tests and observation counts is provided in Table S1. As general principles, for all comparisons of two groups of unpaired samples, we used the Wilcoxon Rank-Sum (Mann-Whitney U) test. For all comparisons of two groups of paired samples, or to compare one group’s median to zero, we used the Wilcoxon Signed-Rank test. For all comparisons of two distributions of samples, we used the Kolmogorov-Smirnov two-sample test. For all analyses of groups of samples over stages of learning, we used an ANOVA evaluated on an ordinary least squares regression of the samples to the learning stage.

#### Plasticity simulations

Each imaged PkC dendrite reported activity of that PkC’s CF input. Imaged PkCs also likely received input from most imaged GrCs, given our small imaging field. Classical GrC→PkC LTD depends only on relative activity of the CF and GrC input, to determine whether that GrC input synapse is weakened at each point in time. We reasoned that, even without access to all GrC inputs to a PkC, we could compute predicted changes in synaptic weight for all imaged GrCs based on simultaneous CF spiking. For simplicity, we assumed that every PkC in our small field of view received input from all imaged GrCs, although in reality the proportion would be lower^54,105^.

The central question of our study was how persistent reward-evoked CF spikes can set meaningful GrC→PkC synaptic input strengths. Thus, we computed predicted LTD caused by CF spikes following reward within [0 0.25] s. It remains unknown to what degree spontaneous 1 Hz background CF spikes—which far outnumber evoked CF spikes—play a similar role in driving synaptic changes, and we considered this broader question beyond the scope of the current study. We thus restricted our analysis to CF reward spikes, except in Figure S5J when we considered alternative inclusion windows. Similarly, we focused on CFs with detectable reward-evoked spiking, whose trial-averaged activity was higher after than prior to reward delivery (70% of all 6,439 CFs, spread across all 117 sessions in 20 mice).

To predict GrC→PkC LTD from reward-evoked CF spikes, for each session, each PkC, and each GrC input, we computed a “running tally” of that GrC input’s LTD events. For every CF reward spike, we tabulated the GrC’s mean activity during the preceding plasticity window [-150, -25] ms. This number—GrC activity just prior to CF reward spike—was taken to be the LTD event magnitude. To bound LTD magnitudes in a defined range [0,1], we applied a logistic function to each LTD magnitude ($\frac{1}{1+e^{-F/s}}$; F=avgGrCsignalinLTDwindow; *s* = 95^th^ percentile fluorescence value per cell).

To summarize the LTD for each GrC, we averaged LTD events across trials and across timepoints. This yielded a single GrC weight vector for each PkC, size N_GrC_-by-1. In reality, opposing and homeostatic mechanisms likely maintain each PkC’s overall synaptic drive in a physiological range. To account for without modeling these unobserved factors, we simply normalized each PkC’s GrC weight vector to unit sum.

Finally, we subtracted the mean of this vector. This was necessary: taking a strictly positive weighted sum of cells will generally produce an output that looks approximately like the average of the population. While GrC→PkC synapses are, in reality, strictly excitatory, an effect similar to mean-subtraction is likely achieved biologically by interneuron networks at the PkC output layer. Finally, given CF uniformity in our small imaging fields, we averaged GrC weight vectors across PkCs. This resulted in a single GrC weight vector per session, which we negated to account for the sign of LTD. These vectors gave rise to analyses in Figure 5; Figure S5.

To predict how GrC weights might affect PkC output, we used a minimal readout: a weighted sum of GrCs, with weights defined above. The resulting weighted sums produced analyses in Figure 6, Figure S5.

Finally, we computed three control weighted sums. First, we computed a simple average across GrCs (uniform weights, e.g., Figure 6C). Alternatively, (brown in Figure 6), we simply randomly reordered the LTD weight vector above. Each GrC thus had a random weight from the true distribution of LTD weights. In the final control, we repeated the entire above LTD procedure but instead using randomly reordered GrC data. Specifically, for every trial and for every GrC, we randomly permuted the timepoints in the trial. Thus, every GrC maintained the same distribution of fluorescence values, but its timing was random with respect to the CF reward spikes. In each case we then repeated the same analyses applied to the true data.

Parameters include: CF reward spike window ([0 0.25] s); cutoff for included PkCs (average activity [0 0.25] s > [−0.3 −0.025] s); LTD window ([−0.15 −0.025] s); logistic function bounding LTD event magnitudes.

All simulation panels operated based only on LTD described above, except Figure S5K which explicitly modeled LTP. To simulate LTP, for each CF we defined a threshold as the bottom 0.5% of all timepoints by firing rate, across trials. This threshold was empirically chosen to yield a total number of LTP events comparable to the total number of LTD events. Each LTP event used the same LTD “eligibility window” of [−150, −25] ms to measure preceding GrC activity. As for LTD, we then tabulated each GrCs activity in each LTP window to be the corresponding LTP event magnitude. The net plasticity effect was the sum of LTD and LTP event magnitudes.

## Supplementary Material

1

2 Table S1 | Summary of statistics, Related to Figures 1–7, S1–S7

3 Video S1 | Example simultaneous GrC-CF two photon imaging, Related to Figure 1
Left, GrCs; Right, CFs, both shown 6x temporally downsampled and played at 4x real-time speed.

4 Video S2 | Example behavior during 1s-to-2s relearning, Related to Figure 2
Left, 1-s-delay expert; Right, 2-s-delay expert, both showing the same mouse on omitted-reward trials aligned to movement onset played at half-speed.

5 Video S3 | Example behavior during PCP2-Ai32 Lobule VI stimulation, Related to Figure 2
Left, omitted-reward laser-off trials; Right, omitted-reward laser-on trials, both aligned to expected reward time with red and blue squares indicating expected reward delivery times and laser activations respectively.

## Figures and Tables

**KEY RESOURCES TABLE:** 

REAGENT or RESOURCE	SOURCE	IDENTIFIER
Antibodies		
GFP	Aves Labs	GFP-1010
RFP	Abcam	62341
Alexa 488 Goat anti-Chicken IgY (H+L)	Thermo	A-11039
Alexa 594 Donkey anti-Rabbit IgG (H+L)	Thermo	A-32754
Bacterial and virus strains		
AAV-L7–6-R-CaMP2	Inoue et al 2015; Nitta et al 2017	
AAV-ef1a-jGCaMP8f	Addgene	Cat# 176756
		
Biological samples		
		
Chemicals, peptides, and recombinant proteins		
Isoflurane	Henry Schein	CAS 26675–46-7
DAPI mounting medium	Southern Biotech	0100–20
Tribromoethanol		
C&B Metabond Quick Adhesive	Parkell	UN1247
		
Critical commercial assays		
		
Deposited data		
		
Experimental models: Cell lines		
		
Experimental models: Organisms/strains		
Mouse: Math1-Cre	Jackson Labs	Stock# 011104
Mouse: Ai93 (TITL-GCaMP6f)-D	Jackson Labs	Stock# 024103
Mouse: ztTA	Jackson Labs	Stock# 012266
Mouse: PCP2-Cre	Jackson Labs	Stock# 004146
Mouse: Ai32	Jackson Labs	Stock# 024109
Mouse: Wild-type CD1	Charles River	Stock# 022
		
Oligonucleotides		
		
Recombinant DNA		
		
Software and algorithms		
MATLAB	Mathworks	https://www.mathworks.com
NoRMCorre	Simons Foundation/Flatiron	https://github.com/flatironinstitute/NoRMCorre
ScanImage	MBF Biosciences	https://www.mbfbioscience.com/products/scanimage/
LabVIEW	National Instruments	http://www.ni.com/en-us/shop/labview.html
PCA/ICA	Mukamel et al	https://github.com/mukamel-lab/CellSort
Kilosort 2.5	Pachitariu et al	https://github.com/MouseLand/Kilosort
phy	Rossant et al	https://github.com/cortex-lab/phy
ecephys	Siegle et al	https://github.com/AllenInstitute/ecephys_spike_sorting
DeepLabCut	Mathis et al	https://github.com/DeepLabCut/DeepLabCut
cNMF	Simons Foundation/Flatiron	https://github.com/dylkot/cNMF
		
Other		
Laser scanning confocal microscope	Zeiss	LSM 510
Stereotaxic Instrument	Kopf	Model 940 Small Animal
Micro syringe pump injector	World Precision Instruments	UMP3T-1
Two-axis robotic manipulandum	Wagner et al 2020	https://github.com/mjwagner/haptic-for-mice
488 nm laser	Coherent	OBIS LX
920 nm + 1064 nm laser	Spark	Alcor 920/1064 2W Dual
Resonant-linear galvo	Avantor	6215H, CRS 8k or 12k
Ti:Sapph tunable laser	Spectra Physics	MaiTai
Focus tunable lens	Optotune	EL-10–30-CI-NIR-LD-MV, EL-16–40-CI-NIR-LD-MV
Fiberport	Thorlabs	PAF2–7A
Steel multimode patch cable	Thorlabs	MR83L01

## Highlights

- First simultaneous recordings of cerebellar granule cells and climbing fibers
- Mice learn reward timing during operant conditioning, with cerebellar contributions
- Sustained granule cell firing links action to future climbing fiber reward spiking
- Granule cell-climbing fiber activity is suited to learning to track long intervals
