# Supplementary material for: A cerebellar granule cell–climbing fiber computation to learn to track long time intervals
Source: Neuron. Author manuscript; Available in PMC 2024 Aug 24. (PMC11343686; doi:10.1016/j.neuron.2024.05.019)
Supplement: 1 [file NIHMS2003659-supplement-1.pdf]

Inventory of Supplemental Information

Figure S1, Related to Figure 1

Figure S2, Related to Figures 2–4

Figure S3, Related to Figures 3, 4

Figure S4, Related to Figure 5

Figure S5, Related to Figures 5, 6

Figure S6, Related to Figure 7

Figure S7, Related to Figure 7

Supplemental References

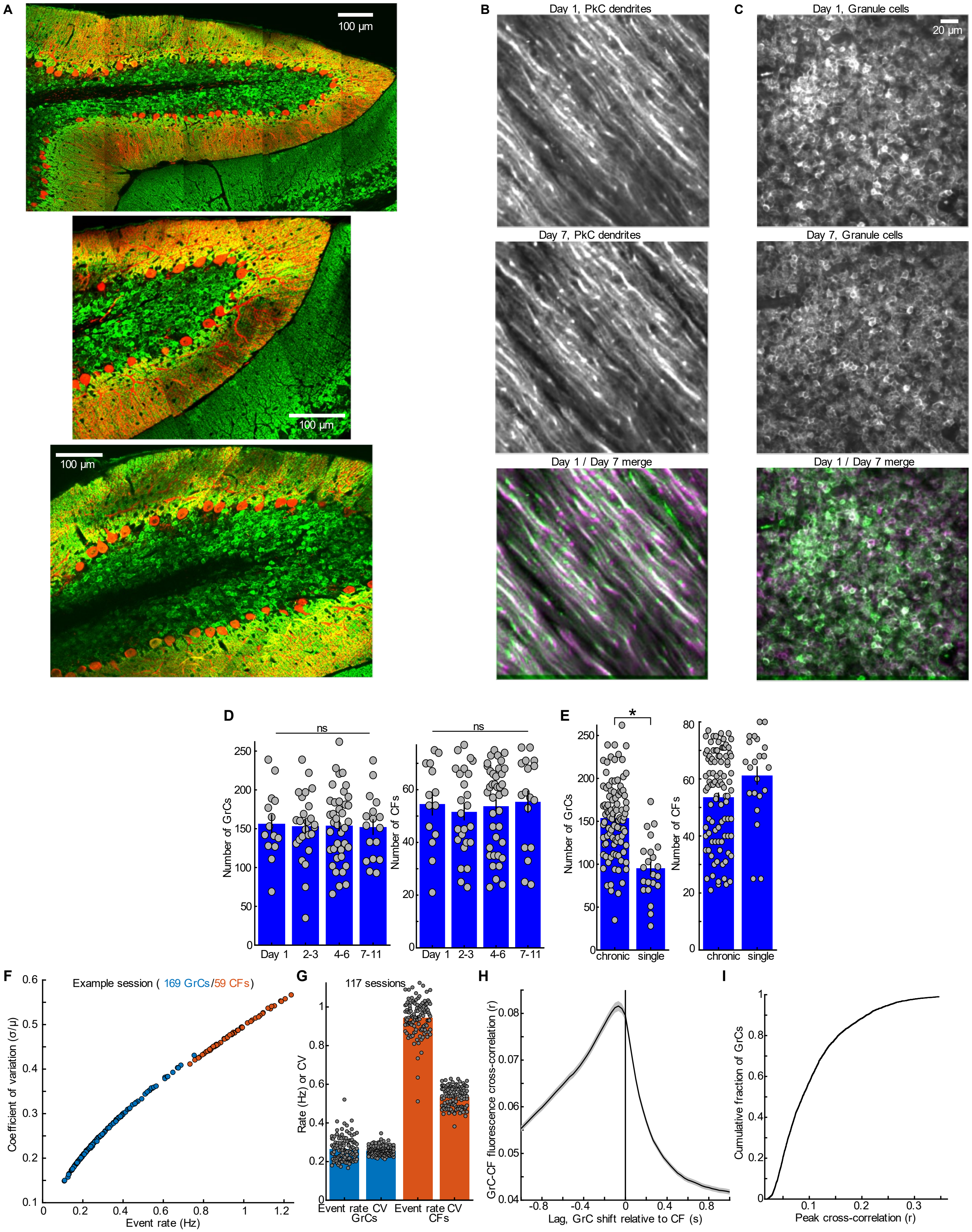

Figure S1 | GrC-CF imaging characterization, Related to Figure 1

(A) As in **Figure 1B**, three additional example histological confocal images with antibody staining showing transgenic GrC expression of GCaMP6f and viral PkC expression of R-CaMP2.

(B,C) Example Day 1 v Day 7 *in vivo* mean two-photon image registration of GrCs and PkC dendrites.

(D,E) Cell counts. (**D**) Number of detected cells did not change with learning ($p=0.8$ and 0.7, 14, 25, 39, and 17 sessions respectively). (**E**) Detected cell counts comparing sessions in which we tracked cells across days ("Chronic", by "importing" cells detected on other days), versus single-shot imaging fields in expert animals ("Single"). GrC counts were higher on chronic sessions ($p<10^{-6}$) while CF cell counts did not differ substantially ($p=0.05$; 95 chronic and 22 single shot sessions, 20 mice).

(F,G) Event rates and coefficients of variation in event rates computed across entire recording sessions (~15-20 minutes), shown as rates/CV of each individual cell in a single imaging session (**F**, 169 GrCs, 59 CFs), or mean rates/CV across cells for each imaging session (**G**, 117 sessions).

(H,I) To quantify gross relationships between activations of GrCs and CFs, we computed cross-correlation functions between the fluorescence of each GrC and the CF population mean over entire recording sessions. The cross-correlation function (**H**, 3,965 GrCs) exhibited a peak just before zero and an asymmetry where GrC activity was more correlated with CF activity prior to CF spikes compared to after. Peak cross-correlation coefficients (**I**) were generally modest.

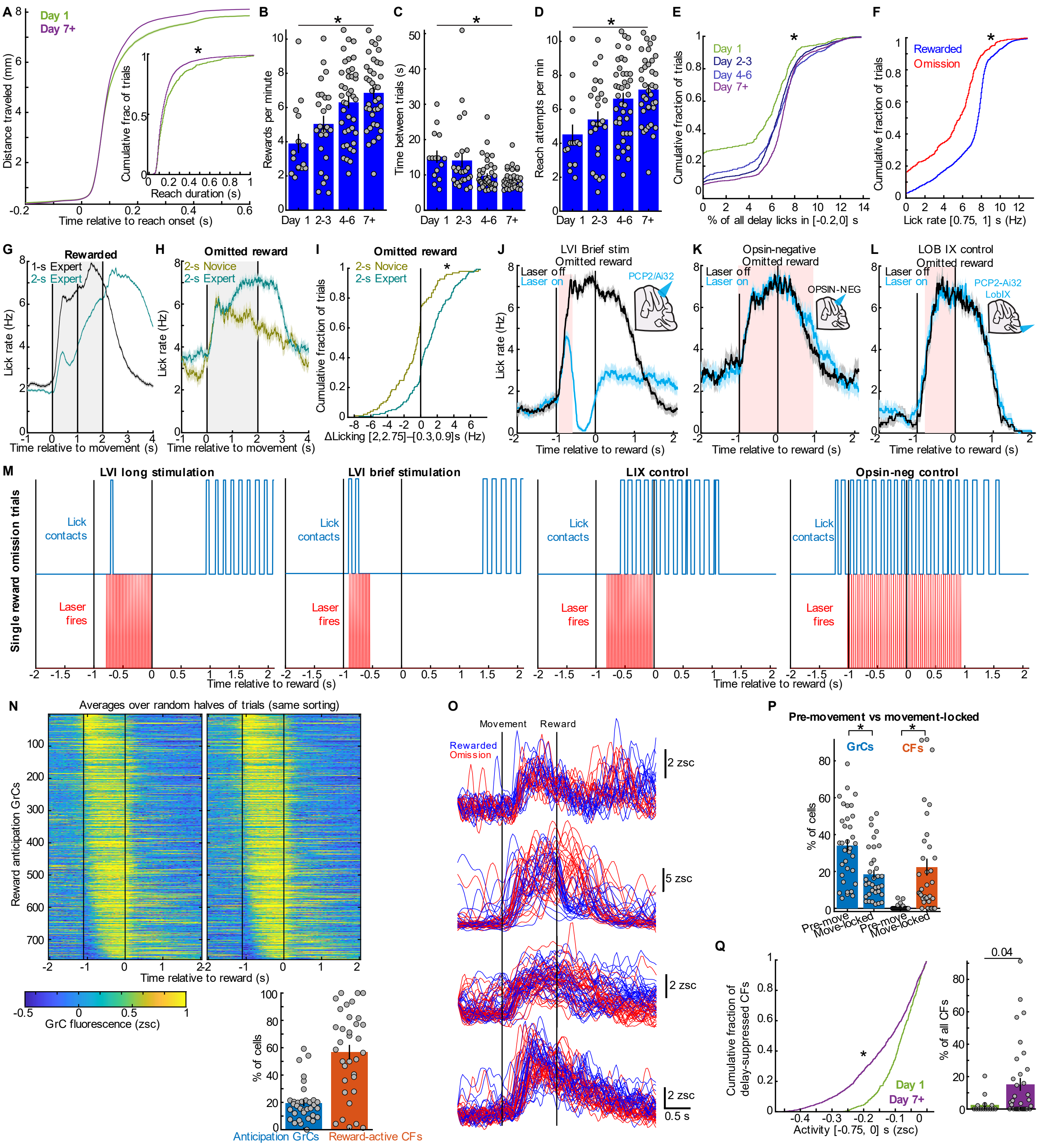

Figure S2 | Behavioral learning, its cerebellar contributions, and neural responses, Related to Figure 2–4

(A) Average forelimb movements recorded by the movement of the manipulandum on Day 1 and Day 7+, aligned to reach onset (1,140 Day 1 and 4,871 Day 7+ trials). Inset, duration decreased with learning ($p<10^{-6}$ KS).

(B-D) Behavior improved by multiple parameters (Learning days/session counts: Day-1/15, Day2-3/25, Day4-6/40, Day7+/37; 20 mice; p-values: 3×10^{-6}, 2×10^{-5}, 10^{-5}).

(E) Fraction of delay licks occurring between [-0.2, 0] s increased with learning ($p<10^{-6}$; 400, 919, 1736, and 2065 trials).

(F) Mice licked significantly more following reward than reward omission ($p<10^{-6}$, 1,570 and 495 trials)

(G) As in **Figure 2F** but for rewarded trials (1222 1-s-expert and 2008 2-s-expert trials, 14 mice, 16 sessions each). These and all subsequent vertical lines denote forelimb movement and reward times and grey regions denote delay periods.

(H-I) For subset of 1-s-to-2-s learners with novice licking data, comparison of reward omission licking in novices and experts (**H**, 97 novice and 155 expert trials, 5 mice). **I,** ΔLick rate during expected reward delivery time vs early in the delay increased with learning ($p<10^{-6}$).

J-L, Further optogenetics data and controls. Quantified in **Fig. 2K**.

(J) PCP2/Ai32 mice received brief PkC stimulation early in the delay (mean: [-0.95, -0.6] s laser-on) on half of reward omission trials (1 in 9 trials; laser-off: 87, laser-on: 85 trials, 7 mice/sessions). Following brief stimulation, licking was disrupted for the remainder of the trial.

(K) Opsin-negative mice with LVI cranial windows received laser illumination (~15 mW) throughout the delay and consumption period (mean laser-on period: [-1,0.9] s; reward omission trial counts: 48 laser-off, 39 laser-on; 3 mice). Licking was similar with and without blue light.

(L) Some PCP2/Ai32 mice underwent a session in which cerebellum Lobule IX was exposed and subjected to blue light illumination on half of reward omission trials during the delay (mean: [-0.8,0] s laser on period; laser-off: 33, laser-on: 34 reward omission trials from 3 mice). Licking was similar with and without blue light.

(M) From left to right, example reward omission trials from the averages shown respectively in **Figure 2J, S2J,L,K**. Raw binary lick sensor contacts (top) are shown in blue, while raw binary laser fire TTL pulses (bottom) are shown in red.

(N) Top, As in **Figure 3G**, here trial-averaged anticipatory GrC profiles are computed on random halves of trials, but sorted as in **Figure 3G**, showing robustness to trial subset selection. Bottom, cells in **Figure 3G,H** shown as percentages of all cells for each of 37 expert sessions from 20 mice.

(O) For four example cells with varied kinetics, from four different mice, from the set of anticipatory GrCs in **Figure 3G**, curves show single-trial fluorescence aligned to reward on rewarded and omission trials (rewarded/omitted trial counts from top: 20/14; 23/23; 31/31; 28/28. Rewarded randomly subsampled to omission trial counts to a minimum of 20).

(P) Prevalence of forelimb movement-activated GrCs (blue) and CFs (orange). Criteria: "Pre-movement," positive activity during [-0.5 -0.25] s with $p<0.05$ and magnitude >0.2 zsc; "Movement-locked," activity during [0 0.2] s >0 and >activity during [-0.3 -0.03] s, with $p<0.05$ and magnitude >0.2 zsc. For GrCs, pre-movement was more common than movement-locked ($p=0.001$), while for CFs we found the reverse ($p<10^{-6}$). Percentages of 3,965 and 1,964 total GrC and CFs.

(Q) Average activity of CFs that were suppressed during delay became more negative with learning ($p<10^{-6}$, CFs with negative activity [-0.75, 0] s, 401/Day1 and 1,178/Day7+ CFs). Inset, CFs significantly suppressed became modestly more common (criteria: >0.2zsc suppression and $p<0.05$; Day 1 vs 7+ $p=0.04$, 15/Day1 and 34/Day7+ sessions).

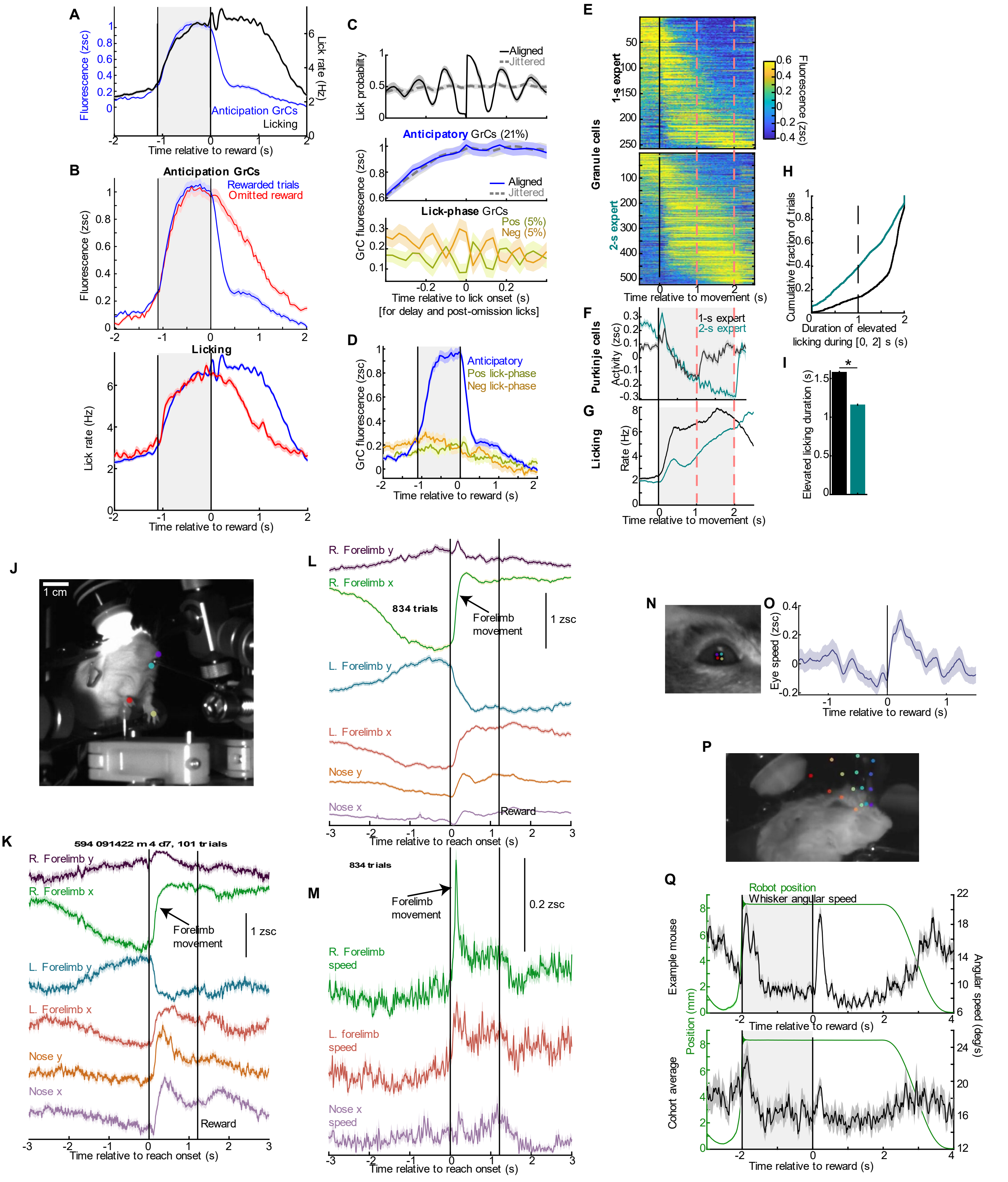

Figure S3 | Learning yields GrC and PkC signals inconsistent with licking, body, eye or whisker movements *per se*, Related to Figure 3, 4

(A-D) Anticipation GrCs qualitatively differ from licking. **A,** Comparison of the activity of reward anticipation GrCs (set defined in **Figure 3G**) with the lick rate, both on rewarded trials. While the two signals rise together prior to reward, GrC activity ceases after reward delivery while licking further increases to its highest values. **B,**

Comparison of reward anticipation GrCs (top) and lick rate (bottom) on rewarded vs omitted reward trials. Whereas GrC activity is higher and prolonged following reward omission vs reward, licking is lower and briefer. **C,D,** To determine whether anticipatory GrCs (**Figure 3G**) were consistent with sensorimotor licking signals, we computed "lick-triggered averages." Based on the results in **A,B,** for a more stringent test we included only licks prior to reward. For every lick onset during the delay, we aligned licking (**C** top**,** 11 mice in 18 sessions) and anticipatory GrC fluorescence (A middle**,** 365 cells) to lick onset, before averaging across all delay licks. As a null hypothesis, we "jittered" lick onsets via uniform random offsets between [-150,+150] ms. Anticipatory GrC fluorescence was unaffected by lick-onset-jittering, demonstrating that these cells are not modulated by lick onset (**C** middle dashed). As a positive control, we identified GrCs with lick-aligned fluorescence weakly but significantly modulated by average onset-aligned licking in **C** (**C** bottom; $r>0.5$ or $r<-0.5$ at $p<0.05$, 85 and 89 GrCs; signals were uncorrelated with brain motion: $|r|=0.08\pm0.02$). (**D**) Trial-averaged fluorescence of each GrC group: unlike anticipatory GrCs, lick-phase-modulated GrCs had no anticipatory buildup.

(E-I) GrC and PkC differences between 1-s-delays versus 2-s-delays were inconsistent with corresponding licking differences. 1-s-vs-2-s expert comparison of GrC activity (**E**), PkC activity (**F**)**,** and lick rate (**G**). 2-s-expert GrC and PkC activity ramped for longer than 1-s-expert activity, with the largest difference in the [1,2] s period—when 2-s-expert licking was lower than in 1-s experts. This results from 1-s experts consuming reward during [1,2] s, while 2-s experts postpone peak licking until late in the delay. **H,I** histograms and bars of the duration of elevated licking during [0, 2] s (compared to mean pre-movement licking [-0.8, -0.3] s; $p<10^{-6}$ 1,222 and 2,008 trials from 14 mice/16 sessions). Thus, changes in GrC and PkC activity between 1-s and 2-s conditions were unexplained by corresponding changes in licking outside of periods of reward anticipation.

(J-Q) We next compared anticipatory GrC signals (**A,B**) to body (**J-M**), eye (**N,O**), and whisker (**P,Q**) movements to determine whether there were selective delay period motor programs consistent with anticipatory GrC activity patterns.

(J) Video frame of mouse body with body coordinates tracked by deeplabcut (DLC) superposed as colored dots.

(K-M) Average trajectories of *x* and *y* camera coordinates of 3 points on the body in an example mouse (**K,** 101 trials) and averaged across all sessions (**L**, 834 trials in 9 expert sessions from 5 mice). Significant body movement accompanied reach onset, but we did not identify body movements selective to the delay period. Alternatively, the time-varying speed of each body coordinate (**M**) exhibited similar trends.

(N,O) We performed eye tracking analysis in expert mice without imaging (**N**). For each of 4 points on the pupil tracked with DLC in each of 3 mice, we computed the speed as a function of time, and then z-scored the values per-point and per-animal. We then computed grand averages across the 4 points and 3 animals (**O**, 125 trials). Arguing against the possibility that widespread GrC anticipation drives or is driven by eye movements, eye movement speed was low prior to expected reward. By contrast, eye movement speed rose substantially after reward delivery.

(P,Q) For each of 6 mice, we tracked 4 points on each of 4-5 whiskers with DLC in an expert 2-s-delay session (example image in **P**). We computed a mean whisker angle for each frame as the average from the whisker base to each of the 3 other tracked points, computed the derivative to yield a time-varying whisker angular velocity, and then the absolute value to yield an angular speed (**Q**; example session, top, 78 trials; cohort average, bottom, 267 trials, 6 sessions/mice). Elevated levels of whisker movement were often seen: when the robot returned the handle to the animal; when the mouse pushed the handle; and when the reward was delivered. By contrast, the delay period featured relatively less whisker movement, which was thus qualitatively distinct from anticipatory granule cell profiles. This does not exclude that such movements are represented in other cerebellar neural features as previously found[1].

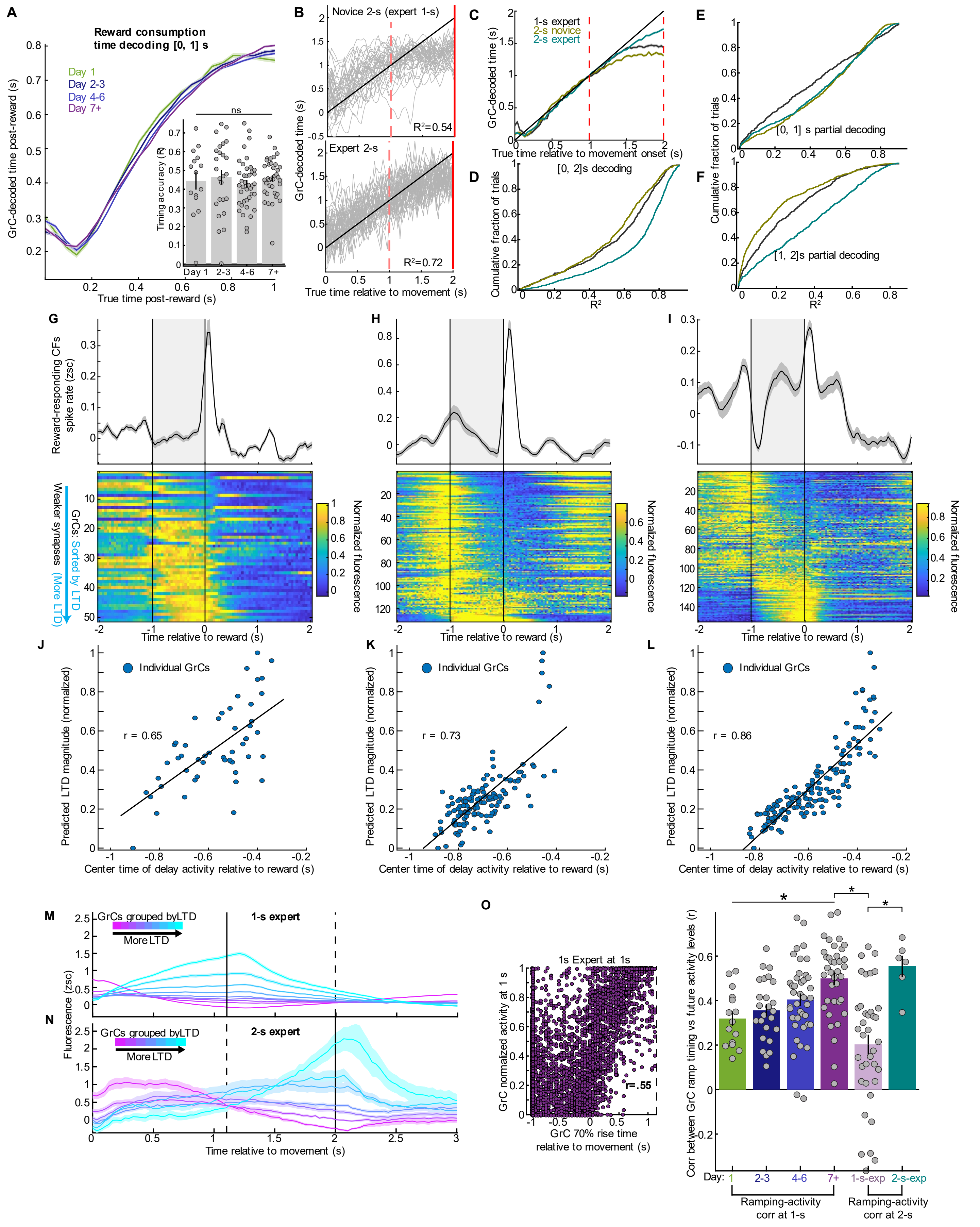
A
Reward consumption time decoding [0, 1] s
Day 1
Day 2-3
Day 4-6
Day 7+
ns
Timing accuracy (R)
GrC-decoded time post-reward (s)
True time post-reward (s)
B
Novice 2-s (expert 1-s)
R²=0.54
Expert 2-s
R²=0.72
GrC-decoded time (s)
True time relative to movement (s)
C
1-s expert
2-s novice
2-s expert
GrC-decoded time (s)
True time relative to movement onset (s)
[0, 2]s decoding
D
Cumulative fraction of trials
R²
E
[0, 1] s partial decoding
F
[1, 2]s partial decoding
G
Reward-responding CFs spike rate (zsc)
Weaker synapses
GrCs: Sorted by LTD
(More LTD)
Normalized fluorescence
Time relative to reward (s)
H
I
J
Individual GrCs
r = 0.65
Predicted LTD magnitude (normalized)
Center time of delay activity relative to reward (s)
K
r = 0.73
L
r = 0.86
M
GrCs grouped by LTD
More LTD
1-s expert
N
2-s expert
Fluorescence (zsc)
Time relative to movement (s)
O
1s Expert at 1s
GrC normalized activity at 1 s
r=.55
GrC 70% rise time relative to movement (s)
Corr between GrC ramp timing vs future activity levels (r)
Day: 1 2-3 4-6 7+ 1-s-exp 2-s-exp
Ramping-activity corr at 1-s
Ramping-activity corr at 2-s

Figure S4 | Reward LTD is sensitive to GrC anticipatory timing throughout the delay, Related to Figure 5

(A) As in **Figure 5A-D**, here regression was used to estimate time during reward consumption as a positive control. Time decoding [0, 1] s from reward was already accurate in novices and did not further increase ($p=1$). Inset**,** average across learning of the GrC-decoded reward consumption time.

(B-F) We used optimal linear decoding on the 2-s delay retraining data to decode time [0, 2] s from forelimb movement. **B**, example decoding for one mouse in the novice 2-s delay session and the expert 2-s delay session (40 randomly chosen trials shown for each for visual clarity). **C,** Average decoding output in all three conditions (606, 625, and 750 trials respectively). **D-F,** Decoding accuracy across all trials was significantly higher in expert sessions (**D**, $p<10^{-6}$, 625 and 750 novice & expert trials). Most decoding improvement in 2-s experts was due to the improvement during [1, 2] s rather than [0,1] s (**E**,**F**; $p=0.0002$ for [0, 1] s, $p<10^{-6}$ for [1, 2] s).

(G–I) Example sessions from 3 additional mice as in **Figure 5I**. Top row, trial-averaged z-scored spike rate of all reward-responding CFs used for LTD computation (CF/trial counts: 49/162, 22/65, 33/67; spike-rate [0, 250] ms from reward higher than [-250, 0] ms). Bottom row, GrC trial-averaged fluorescence peak-normalized per-cell to highlight differences in activation timing (GrC counts: 51, 131, 155).

(J-L) Quantifications of correlations between GrC anticipatory center time and predicted LTD for the above sessions in **G–I**, as in **Figure 5J** ($n = 70$, 131, and 97 GrCs respectively).

(M,N) Unnormalized versions of the plots in **Figure 5L,M** with SEM error bars across neurons.

(O) To explore the GrC properties underlying the relationships in **Figure 5**, for each GrC we computed the time at which it first reached 70% of its peak between [-1,1.1] s relative to reward (x-axis). We compared each GrC's 70%-rise-time to its activity level at the end of the delay (1.1 s). The relationship was strong in 1.1-s experts (left), but weaker earlier in learning (right, $p=4.9\times10^{-5}$). Moreover, when extending the analysis window to 2.1 s, the relationship in 1.1-s experts degraded ($p=2\times10^{-6}$). This relationship was rescued in 2-s experts (>1.1-s experts, $p=0.003$). Sessions: Day1/15, Day2-3/25, Day4-6/40, Day7+/37, 2-s-expert/6. 20 mice and 5 mice.

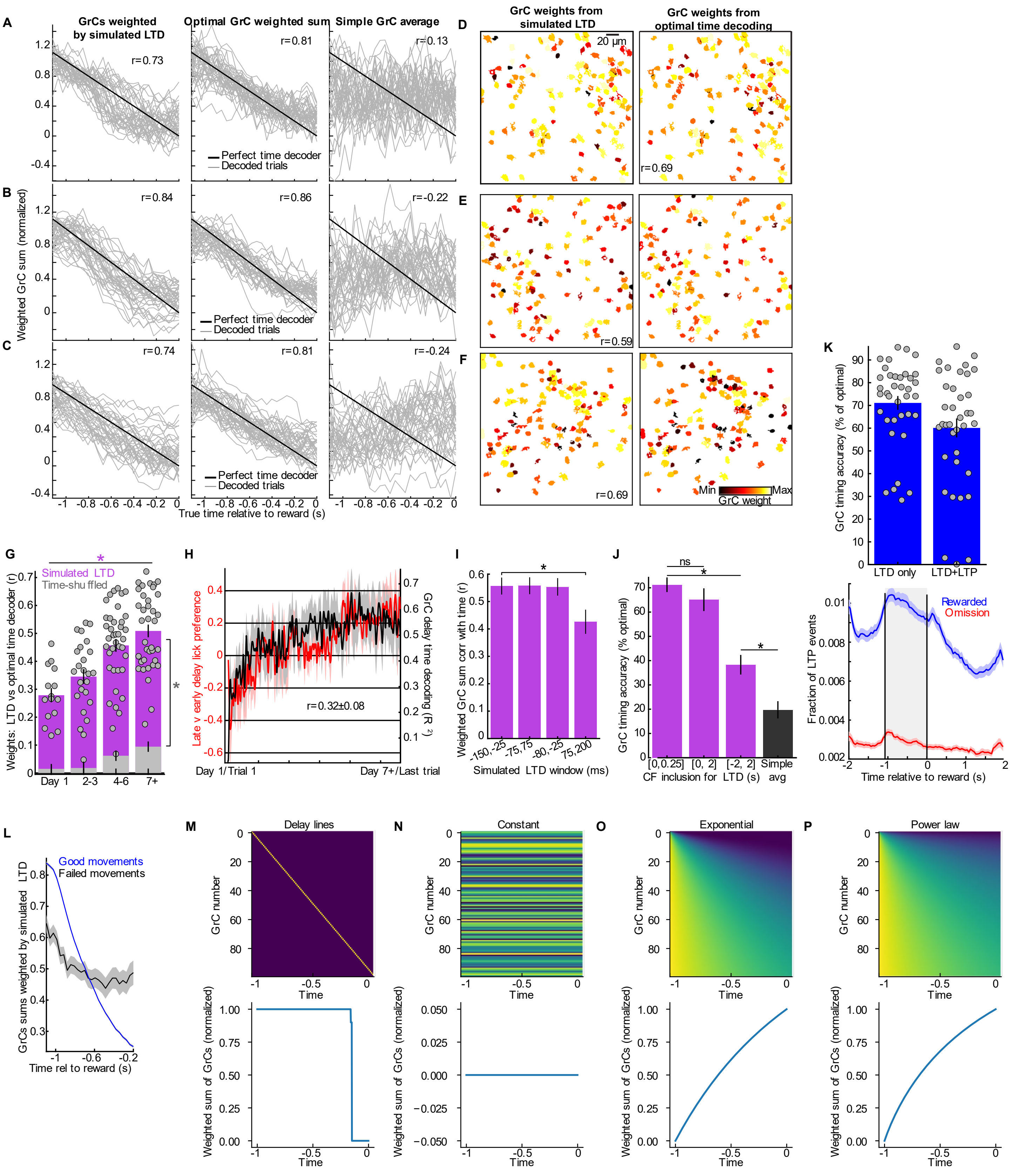

Figure S5 | Simulated-LTD-weighted GrC averages correlated with delay time passage and resembled optimal decoders, Related to Figure 5, 6

(A-C) As in **Figure 6B,C**, Additional example mice comparing predicted PkC output from summing GrCs using synaptic weights derived either from LTD (left column), linear regression onto the time axis (middle column), or

a simple average of all GrCs (right column), with corresponding correlations with time indicated on the graphs (40 trials shown, of 165, 70, and 96 total trials respectively; 83, 135, 123 GrCs, respectively).

(D-F) For example sessions, spatial locations of all detected active GrCs are shown. Each GrC is colored by its readout weight, computed either via LTD (left) or optimal decoding (right). Correlation computed between the two weight vectors ($p<10^{-6}$ for all, GrC counts: 120, 111, 134; trials counts: 153, 68, 186, respectively).

(G) Correlation between weight vectors (e.g., **D-F**) rose with learning ($p<10^{-6}$ 116 sessions). LTD weights computed on time-shuffled GrC data were far less correlated ($p<10^{-6}$).

(H) To compare the trial-wise learning kinetics of behavior and GrC basis changes, we computed a per-trial lick timing preference $(Lick_{late}-Lick_{early})/(Lick_{late}+Lick_{early})$ (Late: [-0.2,0] s; Early: [-0.8,-0.6] s), and the optimal time decoding accuracy (**Figure 5D**) evaluated on that trial. For each mouse with licking and GrC data over learning (7 mice), we concatenated the licking and GrC metrics for all trials from all sessions from that mouse. Finally, we interpolated the resulting vectors to a common number of time points to handle differing trial numbers per mouse, and visualized the average learning curves. Overall, licking and GrC changes proceeded at a similar rate, but the trial-over-trial correlations were only moderate in magnitude (r=0.32+/-0.08).

(I) GrC weighted-sum correlations with time were similar when using a range of LTD windows, consistent with the sustained nature of the GrC signals. However, when using a strictly positive LTD window (i.e., dependent on GrC signals after reward-evoked CF spikes), performance began to degrade ($p=5x10^{-6}$, 37 expert sessions), consistent with the more rapid decay of GrC anticipation following reward delivery.

(J) The simulation aimed to explore the interplay between anticipatory GrCs and reward-evoked CF spiking in canonical LTD. We thus restricted our simulation to reward-evoked CF spikes, which also bypassed open questions about differences in the LTD effects of CF spikes depending on contextual factors (e.g., CF spike bursts have contextually-varying numbers of "spikelets" which change their plasticity impacts[2]; the LTD effect of CF spikes depends on the activity levels of molecular layer interneurons[3]). Alternatively, we examined how the simulation results depended on the temporal range of included CF spikes. Simulations using all post-reward CF spikes were similar to those using only reward-evoked spikes (p=0.13). However, using all pre and post-reward spikes substantially attenuated the magnitude of delay ramps GrC readouts ($p<10^{-6}$), but these still exceeded trivial GrC readout via simple average (p=0.001).

(K) The simulation throughout the manuscript was restricted to LTD. Extensive work demonstrates a symmetric form of LTP evoked by especially low CF spike rates. We implemented a simple LTP triggered by very low CF spike rates (bottom 0.5% of timepoints), such that the frequency of LTP matched the frequency of LTD. The resulting GrC readouts were similar in timing accuracy (**K** top, p=0.05). We then examined the temporal distribution of LTP events (**K** bottom), which demonstrated that the delay period elicited the greatest relative proportion of LTP events, consistent with the moderate suppression of CF spiking during that period and the concurrent highly elevated levels of GrC spiking (20 mice).

(L) Using the GrC weights from LTD computed on normal trials (**Figure 6**), we generated GrC weighted sums on failed trials (length <6 mm) from expert animals. Here, there was little delay timing information, suggesting that animals have little expectation of reward on aborted or failed trials (4,659 normal and 146 failed trials).

(M-P) We considered four different synthetic GrC bases (top). Delay line (**M**) is most similar to the canonical theory of GrC function; constant (**N**) is a trivial negative control; exponential (**O**) and power law (**P**) are two alternative bases with a range of decaying timescales, exhibiting the key property that their activity level around time 0 correlates with their anticipatory timing. We computed an LTD weighted sum for each basis (bottom), via a simulated CF "reward spike" at time 0. Only the exponential and power law bases produced a weighted sum signal that correlated with the passage of time.

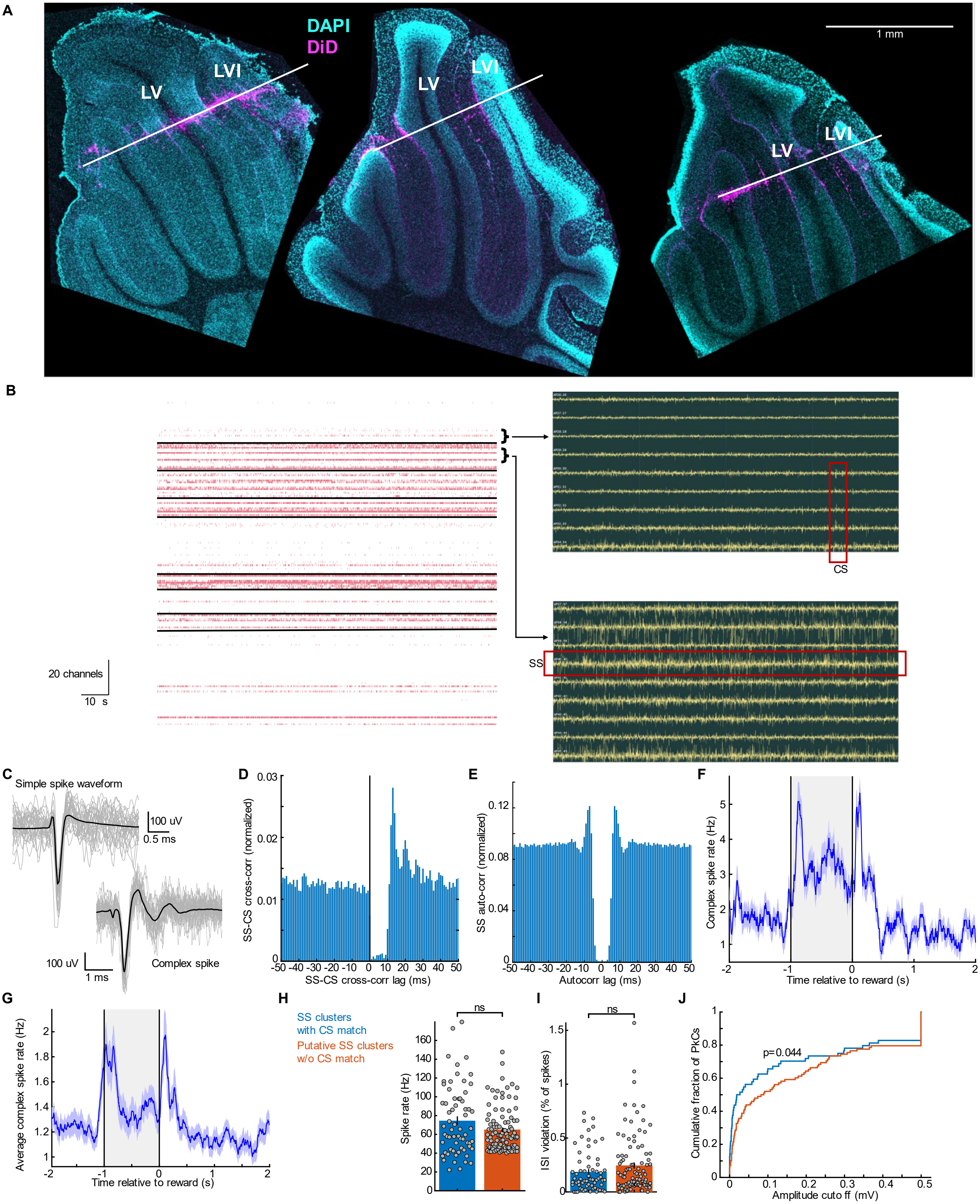

A
DAPI
DiD
LV
LVI
1 mm
B
20 channels
10 s
CS
SS
C
Simple spike waveform
100 uV
0.5 ms
100 uV
1 ms
Complex spike
D
SS-CS cross-corr (normalized)
SS-CS cross-corr lag (ms)
E
SS auto-corr (normalized)
Autocorr lag (ms)
F
Complex spike rate (Hz)
Time relative to reward (s)
G
Average complex spike rate (Hz)
Time relative to reward (s)
H
SS clusters with CS match
Putative SS clusters w/o CS match
ns
Spike rate (Hz)
I
ns
ISI violation (% of spikes)
J
Cumulative fraction of PkCs
p=0.044
Amplitude cutoff (mV)

Figure S6 | Neuropixels recording in posterior cerebellar cortex, Related to Figure 7

(A) Example sagittal histological sections from 3 mice of dorsal posterior cerebellar cortex showing tissue labeled by DiD-coating of the Neuropixels probe. Neuropixels tracks passed mainly through lobule VI, V, and IV. White lines show estimated electrode tracks.

(B) Example spike rasters after Kilosort sorting, mapped onto Neuropixels channel numbers. Horizontal black lines show estimated boundaries (see **H-J**) of likely PkC somatic layers, which were readily distinguished from neighboring layers that either had very little detectable activity (typically the GrC layer and white matter), or very sparse but high magnitude spikes (typically PkC complex spikes detected from the molecular layer).

(C-F) We first identified "confirmed" PkCs based on SS pauses after CS's. Example: characterization of the example expert PkC in **Figure 7A. C,** Waveforms for simple spikes (SS, left) and corresponding complex spikes (CS, right). Traces show 30 example waveforms (grey) and the mean waveforms (black). **D,** Cross-correlogram between detected SS and CS showed the characteristic SS pause after CS (firing rates: 87 Hz and 1.9 Hz). **E,** Autocorrelogram of SS with characteristic mild oscillatory profile. **F,** Trial-averaged CS rate aligned to reward was elevated around movement and reward (124 trials).

(G) Expert trial-averaged activity of all CS units matched to a SS unit by cross-correlogram analysis (as in **F**). Such units showed peaks around movement and just after reward as in the imaging data (64 units).

(H-J) In addition to SS units confirmed by matched cross-correlograms to CS units (C-F) we also detected putative SS units without matched CS clusters. Such SS units were identified based on their proximity to a SS-pause-confirmed PkC (±10 channels/100µm[4]), and high mean spike rates (cutoff: 40 Hz). (**B**). This group of putative SS clusters was similar to confirmed SS clusters by multiple metrics. Mean spike rates (**H**) were not significantly different: 75±5 Hz and 65±2 Hz (p=0.28, 64 and 98 PkCs respectively). Quality metrics including percent of sorted spikes that occurred within 1.5 ms of each other (**I,** ISI violations, p=0.34) and spike sorting amplitude thresholds (**J**, p=0.044) were also both similar between groups. Response profile distributions were also similar (**Figure S7E**). We therefore included both groups of SS units in subsequent analysis.

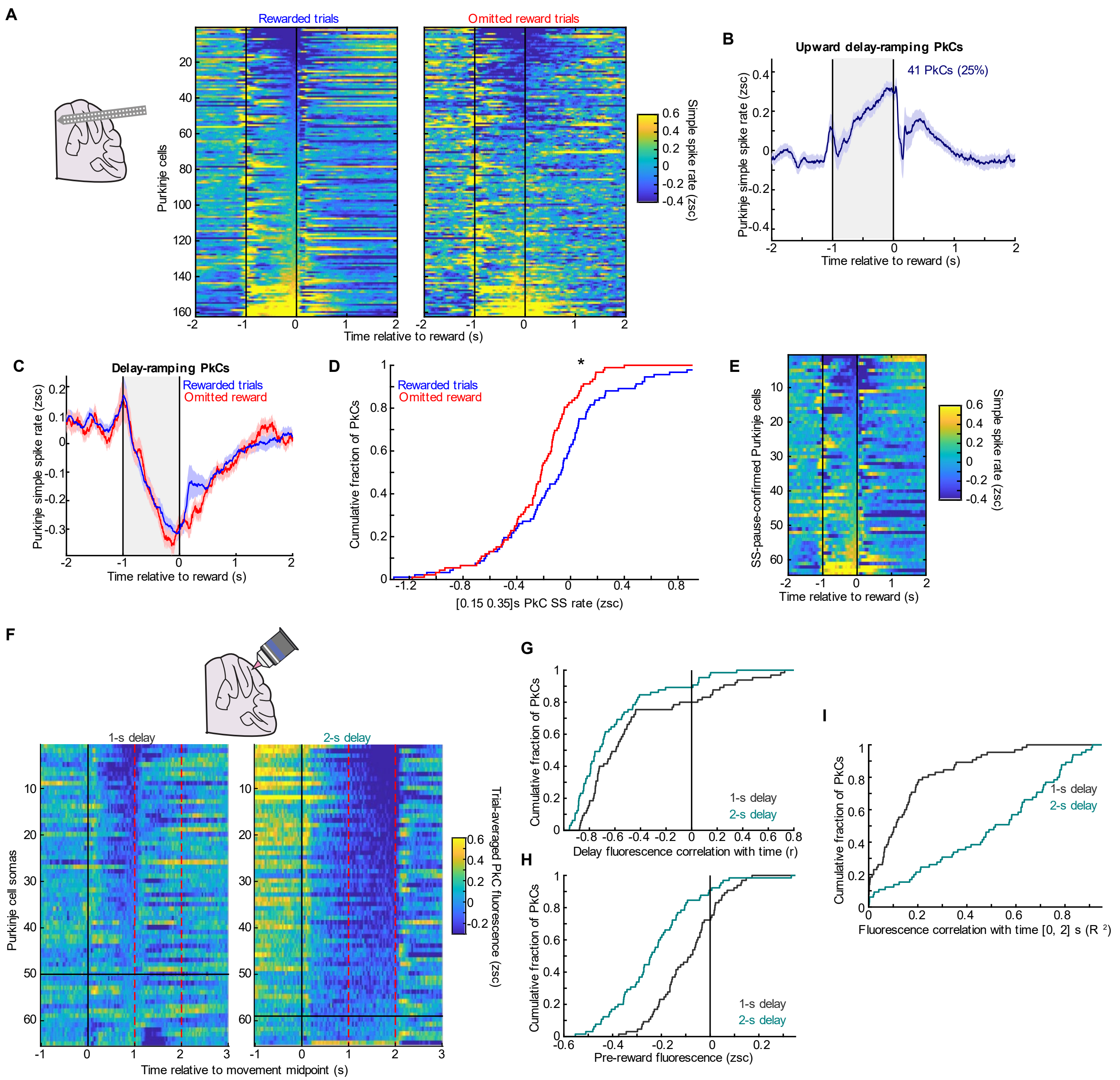

Figure S7 | PkCs ramped with delay passage, Related to Figure 7

(A-E) Further characterization of Neuropixels recordings of PkCs.

(A) Trial-averaged activity of all PkCs in expert recording sessions on rewarded (left) or omitted reward (right) trials, with cells sorted by SS rate just prior to reward ([-150 -25] ms).

(B) Symmetrically to the negative-ramping PkCs in **Figure 7B**, we identified PkCs in expert sessions with positive sloping SS rates that reached rates above baseline just prior to reward, which composed another 25% of all PkCs (41 PkCs). While our minimal LTD simulation on our GrC-CF data does not predict positive ramping, such neurons still carry timing signals. One possibility is that the balance of ramping excitation (from GrCs) and inhibition (from interneurons) might invert the directionality of PkC ramping.

(C,D) For the two groups of expert delay-ramping PkCs in **Figure 7B** and **Figure S7B** we negated the positive-sloping profiles to match their sign to the negative-sloping profiles, and then averaged all such 92 PkCs on rewarded or omitted reward trials (**C**). Such cells returned to baseline faster after reward delivery but extended

their anticipatory response longer following reward omission (**D**, quantified from [0.15, 0.35] s, $p=0.02$). Thus, like the anticipatory GrCs (e.g., **Figure 3**), these PkCs differ from a licking sensorimotor signal *per se*, as licking is far greater following reward than omission.

(E) Like (**A**) but restricted to the set of PkCs matched to a CS unit via SS-pause ($n = 64$), showing grossly similar distributions of response profiles.

(F-I) Further characterization of optical recordings of PkC somas.

(F) Rasters show trial-averaged fluorescence aligned to forelimb movement midpoint at time 0 for all recorded PkC somas in 1-s expert (left) and 2-s expert (right) sessions. Cells are sorted by their fluorescence magnitude just before reward (either [0.9, 1] s or [1.9, 2] s). Vertical black line denotes forelimb movement, while dashed red lines denote 1 s and 2 s. Horizontal line demarcates neurons with negative pre-reward delay activity (top ~80%, left, top ~90%, right) from those with positive activity (1-s: 65 neurons from 4 imaging fields in 2 mice; 2-s: 65 neurons from 3 imaging fields in 2 mice).

(G,H) We quantified each PkC by either its trial-averaged fluorescence correlation with time during the delay (**G**, either [0, 1] s or [0, 2] s) or fluorescence magnitude just before reward (**H**, either [0.9, 1] s or [1.9, 2] s), and then computed cumulative histograms across all PkCs. By either metric, 70-90% of PkCs were negatively modulated by the delay time passage, consistent with our GrC-LTD predictions. Consistent with the longer period of negative ramping, 2-s-expert PkCs exhibited more negative fluorescence just before reward ($p=2\times10^{-6}$).

(I) We computed the correlation with time from [0, 2] s for both 1-s and 2-s sessions as R^2 and tabulated cumulative histograms across all cells. In contrast to the [0, 1] s period, 1-s sessions exhibited no trend for strong correlations when considered over the entire [0, 2] s period, consistent with reward delivery ending the anticipation period and returning PkC activity to baseline, bearing out the prediction from GrC-CF observations and LTD computations ($p<10^{-6}$, **Figure 6G**).

Supplemental References

1. Romano, V., De Propris, L., Bosman, L.W., Warnaar, P., Ten Brinke, M.M., Lindeman, S., Ju, C., Velauthapillai, A., Spanke, J.K., Middendorp Guerra, E., et al. (2018). Potentiation of cerebellar Purkinje cells facilitates whisker reflex adaptation through increased simple spike activity. eLife *7*, e38852. 10.7554/eLife.38852.
2. Yang, Y., and Lisberger, S.G. (2014). Purkinje-cell plasticity and cerebellar motor learning are graded by complex-spike duration. Nature *510*, 529-532. 10.1038/nature13282.
3. Ke, Z., Zhen, Y., Michael, A.G., Garrett, G.G., Don, B.A., and Jason, M.C. (2023). Molecular layer disinhibition unlocks climbing-fiber-instructed motor learning in the cerebellum. bioRxiv, 2023.2008.2004.552059. 10.1101/2023.08.04.552059.
4. Beau, M., Herzfeld, D.J., Naveros, F., Hemelt, M.E., D'Agostino, F., Oostland, M., Sánchez-López, A., Chung, Y.Y., Maibach, M., Stabb, H.N., et al. (2024). A deep-learning strategy to identify cell types across species from high-density extracellular recordings. bioRxiv, 2024.2001.2030.577845. 10.1101/2024.01.30.577845.
